# Framboidal ABC triblock copolymer vesicles: a new class of efficient Pickering emulsifier†
†Electronic supplementary information (ESI) available: Details of the structural model used for SAXS analysis, DMF GPC traces, assigned ^1^H NMR spectra, SAXS data and fittings in aqueous sucrose solution, schematic for three-layer model for SAXS analysis, SEM images of Pickering emulsions, visible absorption spectra and calibration plots for G_63_H_350_B_*z*_ vesicles and two tables summarizing SAXS fitting parameters. See DOI: 10.1039/c5sc02346g


**DOI:** 10.1039/c5sc02346g

**Published:** 2015-08-05

**Authors:** C. J. Mable, N. J. Warren, K. L. Thompson, O. O. Mykhaylyk, S. P. Armes

**Affiliations:** a Department of Chemistry , University of Sheffield , Brook Hill , Sheffield , South Yorkshire S3 7HF , UK . Email: o.mykhaylyk@sheffield.ac.uk ; Email: s.p.armes@sheffield.ac.uk

## Abstract

Pickering emulsions offer important advantages over conventional surfactant-stabilized emulsions, including enhanced long-term stability, more reproducible formulations and reduced foaming problems. The recent development of polymerization-induced self-assembly (PISA) offers considerable scope for the design of a wide range of block copolymer nanoparticles with tunable surface wettability that may serve as bespoke Pickering emulsifiers. In the present study, we exploit PISA to design a series of model framboidal ABC triblock copolymer vesicles with exquisite control over surface roughness. Transmission electron microscopy (TEM) and small-angle X-ray scattering (SAXS) were utilized to characterize these nanoparticles, which were subsequently used to stabilize *n*-dodecane emulsion droplets in water. The adsorption efficiency, *A*_eff_, of the nanoparticles at the *n*-dodecane/water interface was determined as a function of increasing vesicle surface roughness using a turbidimetry assay. A strong correlation between surface roughness and *A*_eff_ was observed, with *A*_eff_ increasing from 36% up to 94%. This is a significant improvement in Pickering emulsifier efficiency compared to that reported previously for similar vesicles with smooth surfaces. In summary, nanoparticles with appreciable surface roughness are much more effective Pickering emulsifiers and this parameter can be readily fine-tuned using a highly efficient PISA formulation.

## Abstract 2

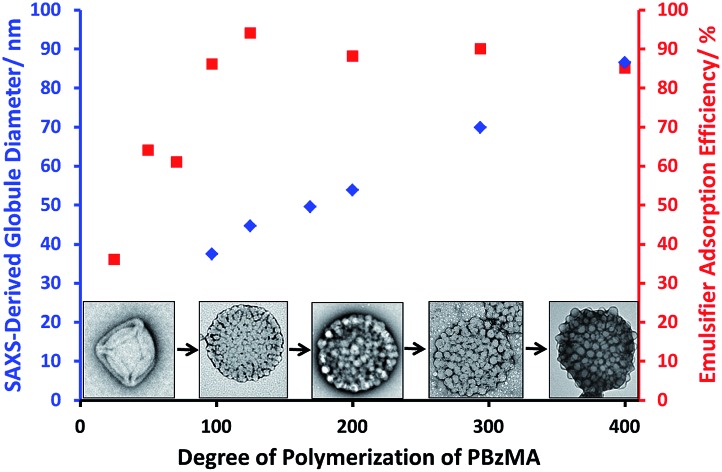
Framboidal triblock copolymer vesicles prepared via RAFT-mediated PISA are characterized by SAXS and TEM; a Pickering emulsifier adsorption efficiency of up to 94% is obtained for a mean globule size of 45 nm.

## Introduction

Pickering emulsions are water or oil droplets that are stabilized by colloidal particles and have been recognised for more than a century.^1^ These systems typically exhibit greater droplet stability compared to surfactant-stabilized emulsions.^2^ This is the result of strong, essentially irreversible particle adsorption at the oil–water interface, which minimizes the interfacial area between the two immiscible liquids and provides a steric barrier towards droplet coalescence.^2^,^3^ A wide range of nanoparticles such as silica sols^4^,^5^ polystyrene latexes^6^–^9^ and inorganic clays^10^ have been shown to be effective Pickering emulsifiers. More recently, cross-linked block copolymer nanoparticles prepared by reversible addition–fragmentation chain transfer (RAFT) polymerization^11^,^12^ have proven to be effective oil-in-water^13^ and water-in-oil^14^ Pickering emulsifiers. For example, Thompson *et al.*^13^ prepared highly stable emulsions using poly(glycerol monomethacrylate-*block*-2-hydroxypropyl methacrylate-*block*-ethylene glycol dimethacrylate) (PGMA-*b*-PHPMA-*b*-PEGDMA) triblock copolymer vesicles. Turbidimetry studies indicated that these nanoparticles had an adsorption efficiency of as low as 57%, depending on the vesicle concentration used for homogenization. This relatively poor adsorption efficiency was in part attributed to the high water content of the vesicles, which leads to a low Hamaker constant compared to solid particles.

In principle, particle wettability can be modulated by increasing surface roughness in order to enhance interfacial adsorption and hence Pickering emulsion stability. This hypothesis has been recently verified by San-Miguel and Behrens, who coated cationic silica microparticles with anionic nanoparticles prepared from a commercial methacrylic acid/methyl methacrylate statistical copolymer (Eudragit S-100; 33% methacrylic acid). Solvent annealing of the nanoparticle coating was used to control the surface roughness of the microparticles, which were subsequently utilized to prepare oil-in-water Pickering emulsions at pH 5.^15^ In a related study, carbon black particles possessing a characteristic fractal morphology were used to stabilize the water/*n*-octane interface.^16^

In the present study, we prepare a series of ABC triblock copolymer vesicles of exquisitely tunable surface roughness.^17^ First, a poly(glycerol monomethacrylate) (PGMA) macromolecular chain transfer agent (macro-CTA) is chain-extended using 2-hydroxypropyl methacrylate (HPMA) via RAFT aqueous dispersion polymerization. *In situ* polymerization-induced self-assembly (PISA) occurs to form nascent nanoparticles comprising poly(2-hydroxypropyl methacrylate) (PHPMA) cores that are sterically stabilized by the water-soluble PGMA chains.^18^–^21^ Depending on the relative volume fractions of the PGMA and PHPMA blocks, well-defined copolymer spheres, worms or vesicles can be obtained at relatively high solids directly in aqueous solution.^22^ The mechanism of formation of the vesicular morphology has been investigated by Blanazs *et al.*^22^,^23^ Chambon and co-workers reported that chain extension of such PGMA–PHPMA precursor vesicles using a water-insoluble monomer such as benzyl methacrylate (BzMA) results in the formation of framboidal (raspberry-like) ABC triblock copolymer vesicles *via* seeded RAFT emulsion polymerization.^24^ Herein, we revisit this formulation in order to gradually increase the target degree of polymerization (DP) of the PBzMA block over a wide range using the same batch of PGMA–PHPMA diblock copolymer vesicles. This systematic approach enables the evolution of the framboidal morphology to be explored in detail: a series of vesicles with gradually increasing surface roughness are produced, as judged by transmission electron microscopy (TEM) and small-angle X-ray scattering (SAXS). These framboidal vesicles are then employed to prepare oil-in-water Pickering emulsions using either *n*-dodecane or *n*-hexane as the droplet phase. The emulsions are characterized in terms of their droplet size distributions and the particle adsorption efficiency at the oil/water interface is assessed as a function of surface roughness. For the sake of brevity, a shorthand notation is utilized throughout the manuscript to describe the various block copolymers. Thus G, H, B, and E denote glycerol monomethacrylate, 2-hydroxypropyl methacrylate, benzyl methacrylate and ethylene glycol dimethacrylate, respectively. For example, G_*x*_H_*y*_B_*z*_ represents a poly(glycerol monomethacrylate-*block*-2-hydroxypropyl methacrylate-*block*-benzyl methacrylate) copolymer, where *x*, *y*, and *z* indicate the mean degrees of polymerization (DP) of the three respective blocks.

## Results and discussion

### Synthesis and characterization

**Fig. 1:**
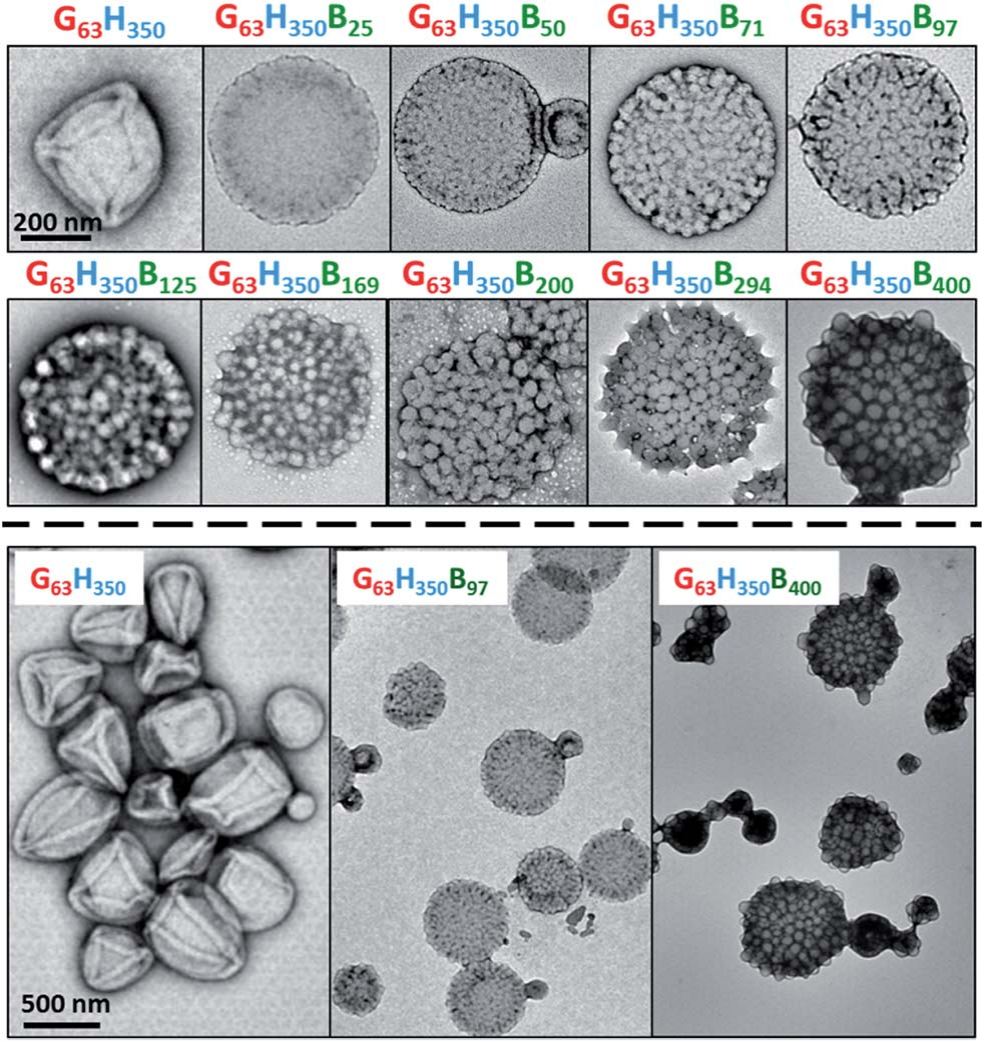
Representative TEM images obtained for a series of framboidal G_63_H_350_B_*z*_ triblock copolymer vesicles (where *z* = 25–400) and also the precursor G_63_H_350_ diblock copolymer vesicles. A 200 nm scale bar applies for the first ten images, while a 500 nm scale bar applies for the last three images.

The initial RAFT solution polymerization of GMA was conducted in ethanol at 70 °C to generate a near-monodisperse G_63_ macro-CTA (*M*_w_/*M*_n_ = 1.16; see Fig. S1† and Table 1). After purification, this water-soluble macro-CTA was utilized for the *in situ* RAFT aqueous dispersion polymerization of HPMA at 15% w/w solids. ^1^H NMR studies indicated that >99% HPMA conversion was achieved within 2 h at 70 °C, as expected from previous studies.^23^ Gel permeation chromatography (GPC) studies indicated that near-monodisperse diblock copolymers were obtained with minimal macro-CTA contamination and high blocking efficiencies (*M*_w_/*M*_n_ = 1.16; see Fig. S1† and Table 1). GPC traces were invariably unimodal but typically exhibited a high molecular weight shoulder. The latter feature is attributable to low levels of dimethacrylate impurity within HPMA (approximately 0.07 mol% as judged by HPLC analysis), which results in light branching of the PHPMA chains. TEM images (see first TEM image shown in Fig. 1) reveal a pure vesicular morphology, as expected for this asymmetric diblock composition. The vesicle folds that are discernible in the TEM images are the result of buckling and/or partial collapse of these relatively delicate nano-structures under the ultrahigh vacuum conditions. These well-defined G_63_H_350_ diblock copolymer precursor vesicles were also characterized by DLS (see Table 1) and then utilized for the *in situ* RAFT seeded emulsion polymerization of BzMA at 70 °C to produce a series of nine G_63_H_350_B_*z*_ triblock copolymers (where *z* ranges from 25 to 400).

**Table 1:** Summary of ^1^H NMR calculated composition and conversion, GPC number-average molecular weight (*M*_n_) and polydispersity (*M*_w_/*M*_n_) and DLS hydrodynamic diameter (*D*_h_) obtained for a G_63_ macro-CTA, linear G_63_H_350_ diblock copolymer precursor vesicles and framboidal G_63_H_350_B_*z*_ triblock copolymer vesicles (where *z* ranges from 25 to 400)

Copolymer composition	Conv. (%)	*M*_n_ (kg mol^–1^)	*M*_w_/*M*_n_	*D*_h_ (PDI) (nm)
G_63_	—	17.6	1.16	—
G_63_H_350_	>99^*a*^	82.2	1.16	362 (0.08)
G_63_H_350_B_25_	100	87.3	1.16	401 (0.09)
G_63_H_350_B_50_	100	100.0	1.10	411 (0.09)
G_63_H_350_B_71_	94	102.1	1.10	406 (0.09)
G_63_H_350_B_97_	97	104.5	1.11	407 (0.07)
G_63_H_350_B_125_	100	112.2	1.12	394 (0.04)
G_63_H_350_B_169_	97	114.3	1.13	364 (0.06)
G_63_H_350_B_200_	100	117.7	1.15	375 (0.08)
G_63_H_350_B_294_	98	130.7	1.18	366 (0.05)
G_63_H_350_B_400_	100	140.9	1.25	418 (0.12)

^*a*^Refers to HPMA conversion in this case.

^1^H NMR studies for these triblock copolymers (see Fig. S2†) indicate conversions greater than 96% (see Table 1). Signal j at 7.1–7.4 ppm, which is assigned to the five aromatic BzMA protons, increases on targeting higher DPs. DMF GPC studies confirmed that near-monodisperse triblock copolymers were obtained (*M*_w_/*M*_n_ ranges from 1.10 to 1.25) with high blocking efficiencies; see Fig. S1† and Table 1. It is noteworthy that these polydispersities are significantly lower than those reported by Chambon *et al.*, who reported *M*_w_/*M*_n_ values as high as 1.50.^24^ This is most likely attributable to the higher macro-CTA/initiator molar ratio of 5.0 employed in the present work. In contrast, Chambon *et al.* used a macro-CTA/initiator molar ratio of just 2.0, which is known to lead to reduced living character for RAFT polymerizations and may also lead to homopolymer impurities.^25^ Moreover, it is worth noting that Chambon *et al.* only targeted three G_58_H_350_B_*z*_ copolymers, for which *z* was 200, 300 or 400.^24^

In the present study, we explore the evolution of the framboidal morphology in much more detail (nine G_63_H_350_B_*z*_ copolymers, with *z* ranging from 25 to 400) while achieving significantly better control over the copolymer molecular weight distribution.

DLS and TEM studies indicate that the vesicle diameter is more or less unchanged as the PBzMA DP is increased (see Table 1 and Fig. 1). TEM analysis of the G_63_H_350_ diblock copolymer precursor vesicles indicates a relatively smooth and featureless surface morphology (see Fig. 1). After chain extension with BzMA, the vesicle surface becomes increasingly rough until individual micelle-like globules of approximately 34 nm can be observed at a block copolymer composition of G_63_H_350_B_97_. This suggests that nano-scale phase separation occurs within the vesicle walls during the polymerization of BzMA, as previously reported by Chambon *et al.*^24^ As the target PBzMA DP is increased, the globules grow in size and prominence.

### Small-angle X-ray scattering (SAXS) studies

**Fig. 2:**
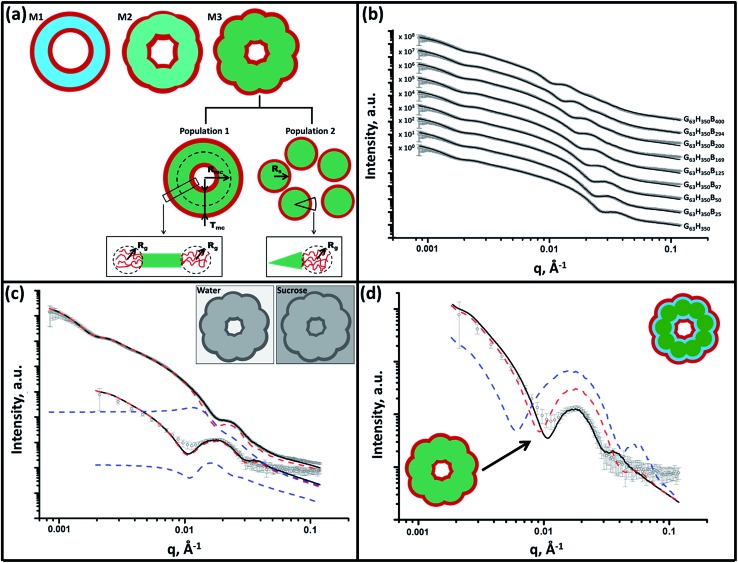
Panel (a) shows a schematic representation of the structural morphology of both a G_63_H_350_ diblock copolymer precursor vesicle and a series of G_63_H_350_B_*z*_ triblock copolymer vesicles, where red = PGMA (G), light blue = PHPMA (H), light green = mixed PHPMA and PBzMA (B) membrane where *z* ≤ 50, and dark green = mixed PHPMA and PBzMA membrane where *z* ≥ 97. Panel (b) shows SAXS patterns obtained for 1.0% w/w aqueous dispersions of G_63_H_350_ diblock copolymer precursor vesicles (*z* = 0) and a series of framboidal G_63_H_350_B_*z*_ triblock copolymer vesicles, where *z* = 25, 50, 97, 125, 169, 200, 294 or 400. Solid lines represent fitting curves: for *z* = 0, 25 or 50, a single population model was sufficient, whereas two populations were required for higher *z* values. For clarity, the SAXS patterns are shifted upward by an arbitrary factor indicated in the figure. Panel (c) displays SAXS patterns obtained for both 1.0% w/w aqueous sucrose and aqueous dispersions of framboidal G_63_H_350_B_125_ triblock copolymer vesicles. Fitting curves are represented by solid black lines. A two-population model was required for fitting [morphology 3 (M3) in panel (a)]: population 1 is represented by red dashed lines and population 2 is represented by blue dashed lines. Inset: schematic representations of the X-ray contrast achieved in both pure water and aqueous sucrose solutions. Panel (d) shows SAXS patterns obtained for a 1.0% w/w aqueous sucrose dispersion of framboidal G_63_H_350_B_125_ triblock copolymer vesicles. The solid black line is the fit obtained for the continuous core model, while the dashed red and blue lines are the fits obtained for a fully phase-separated three-layer model when using *x*_sol_ parameters of 0.50 (red) and 0.34 (blue).

SAXS is used to further characterize this *framboidal* vesicular morphology. TEM images (Fig. 1) suggest three distinct particle morphologies: vesicles with smooth membranes (morphology 1), vesicles with pitted membranes (morphology 2) and vesicles with globular membranes (morphology 3) (see Fig. 2a). The latter morphology is comparable to the polymer core–particulate silica shell particles reported by Balmer and co-workers.^26^–^29^ In this earlier work, Monte Carlo simulations were utilized to demonstrate^26^ that the SAXS patterns obtained for such nanocomposite particles can be described by a two-population model represented by a superposition of two scattering signals originating from a core–shell spherical particle (population 1) and the small spherical silica particles that formed the shell (population 2).

A similar approach to SAXS analysis has been undertaken in the present study. Accordingly, population 1 represents the vesicles and population 2 describes the globules within the vesicle membrane (see Fig. 2a, morphology 3 and ESI for the SAXS fitting model, eqn (S1)–(S10)†). Population 1 of the proposed two-population model corresponds to the initial morphology 1 (smooth vesicles) and is thus appropriate for SAXS analysis of the G_63_H_350_ diblock copolymer precursor. Morphology 1 is well described by the vesicle model (population 1 in eqn (S1)†), which produced a reasonably good fit to the SAXS pattern over six orders of magnitude of X-ray scattering intensity (Fig. 2b, Table S1,† G_63_H_350_). The calculated vesicle radius, *R*_out_, of 176 nm (Table S1†) is consistent with both TEM observations (Fig. 1) and DLS data (Table 1). The mean vesicle diameter is estimated to be 350 nm by TEM analysis, while DLS studies indicate a mean hydrodynamic vesicle diameter (*D*_h_) of 362 nm with a relatively low polydispersity index (PDI) of 0.08. The radius of gyration (*R*_g_) of the G_63_ corona block was determined to be 2.1 nm from model fitting of the G_63_H_350_ SAXS pattern. This experimental value is comparable to a theoretical estimate: the projected contour length of a single GMA monomer is 0.255 nm (two carbon bonds in all-trans conformation), the total contour length of a G_63_ block, *L*_PGMA_ = 63 × 0.255 nm = 16.07 nm and the Kuhn length of 1.53 nm, based on the literature value for poly(methyl methacrylate),^30^ result in an estimated *R*_g_ of (16.07 × 1.53/6)^1/2^, or 2.02 nm. The water volume fraction, *x*_sol_, in the membrane core is approximately 0.50 according to the SAXS data fit. The vesicle model (population 1) also produced a good fit to the experimental SAXS patterns corresponding to the triblock copolymer vesicles containing a relatively short PBzMA block corresponding to morphology 2 (Fig. 2b and Table S1,† samples G_63_H_350_B_25_ and G_63_H_350_B_50_). This result is consistent with TEM observations (Fig. 1), which suggests that such copolymer compositions produce only surface-pitted vesicles that do not significantly affect their membrane structure. However, in order to produce satisfactory fits to SAXS patterns obtained for genuine *framboidal* vesicles prepared by targeting longer PBzMA blocks (*e.g.* G_63_H_350_B_*z*_, *z* = 97–400) incorporation of population 2 (spherical micelles, which correspond to the micelle-like globules) into the model, eqn (S1),† was essential (Fig. 2c, SAXS data corresponding to a continuous phase comprising pure water). A superposition of scattering signals from two populations (vesicles and spherical micelles) used in the model produces good fits to the SAXS data over a wide range of PBzMA block DPs (Fig. 2b and Table S1†).

It is assumed that both the *R*_g_ of the PGMA block and the water content within the hydrophobic vesicles membrane do not change during the growth of the PBzMA block. Thus, the *R*_g_ and *x*_sol_ values obtained for the G_63_H_350_ diblock precursor vesicles were used as fixed parameters for SAXS fitting of the final triblock copolymers. The same batch of PGMA macro-CTA was used for all copolymer syntheses described in this work, so the assumption of a fixed *R*_g_ for this block is perfectly reasonable. At first sight, it is questionable whether *x*_sol_ should remain constant when growing a progressively longer PBzMA block. This is because PBzMA is significantly more hydrophobic than PHPMA, hence a gradual reduction in *x*_sol_ with increasing PBzMA content might be expected. However, the developing framboidal character of the vesicle membrane necessarily leads to the incorporation of additional water molecules (see Fig. S3†). We show below that this feature is sufficient to maintain a constant *x*_sol_, regardless of the PBzMA content of the copolymer. An *x*_sol_ of 0.50 is obtained for the membrane-forming PHPMA block of the precursor G_63_H_350_ diblock copolymer vesicles. This value is consistent with recent work by Warren *et al.*, who reported *x*_sol_ values ranging from 0.38 to 0.66 for G_55_H_*y*_ vesicles when varying *y* from 200 to 1000, respectively.^31^ Assuming additivity, if the PBzMA component has a water content of zero then *x*_sol_ might be expected to decrease from 0.50 for G_63_H_350_ diblock copolymer vesicles to 0.20 for G_63_H_350_B_400_ triblock copolymer vesicles (see Table S2†). Using these *x*_sol_ values as fitting parameters produces comparable results to those obtained when *x*_sol_ is kept constant at 0.50 (see Tables S1 and S2†). This suggests that the SAXS parameters are relatively insensitive to *x*_sol_. However, marginally better fits to the model, especially at high *q*, are obtained when *x*_sol_ is taken to be 0.50, regardless of the copolymer composition. This is most likely because, for population 1 of the SAXS model, it is assumed that water is distributed evenly within the hydrophobic component of the vesicle membrane (see Fig. S3†).

**Fig. 3:**
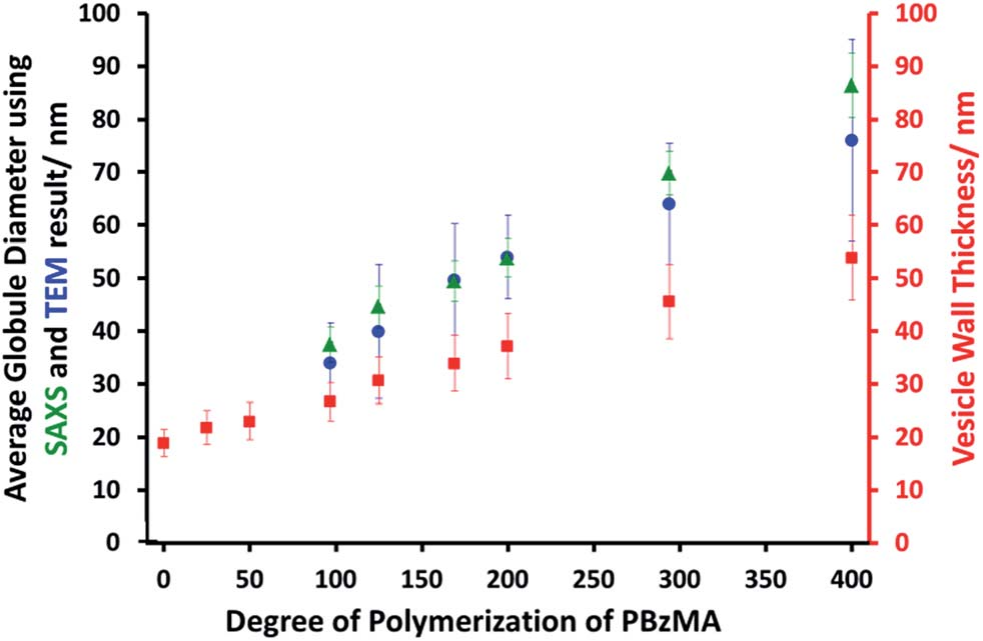
Variation in mean micelle/globule diameter and vesicle wall thickness (*T*_mc_) with degree of polymerization (*z*) for G_63_H_350_B_*z*_ triblock copolymer vesicles obtained from SAXS (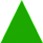, 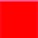) and TEM (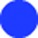) data. [N.B. no mean globule diameters can be determined for *z* = 0, 25 and 50 because these vesicles do not exhibit framboidal character.]

It is true that the overall volume fraction of water associated with the *hydrophobic* block(s) is *reduced* as the diblock copolymer precursor is chain-extended with BzMA. However, the local increase in curvature caused by the growth of the pseudo-spherical globules actually leads to a *higher* volume fraction of water becoming associated with the membrane *as a whole* (see yellow regions in Fig. S3†). This water volume fraction (or *x*_sol_) can be estimated geometrically by calculating the free volume associated with a sphere of radius 0.5*a* placed within a cube of length *a*:
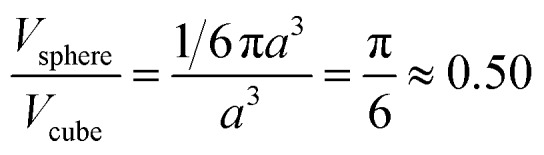


SAXS analysis shows that the thickness of the hydrophobic component of the vesicle membrane (*T*_mc_) increases on targeting higher DPs for the PBzMA block (Table S1† and Fig. 3). However, the overall vesicle dimensions remain virtually constant over all copolymer compositions (*R*_out_ ∼ 174 nm, Table S1†), which is consistent with our TEM observations (Fig. 1) and DLS studies (Table 1). Taken together, these data suggest that the vesicle growth mechanism leads to a gradual reduction in the volume of the vesicle lumen, as reported recently by Warren and co-workers for non-framboidal G_55_H_*y*_ vesicles, where *y* ranges from 200 to 2000.^32^ The nanoscale phase separation that occurs within the vesicle membrane described by the spherical micelle model (population 2) can also be identified from SAXS analysis.

Both the spherical micelle radius (*R*_s_) and the relative concentration of the second population (*c*_2_/*c*_1_) increase at a higher PBzMA block volume fraction, *V*_PBzMA_ (Table S1†). Moreover, the *R*_s_ values are consistent with those estimated from TEM images (Fig. 3). TEM studies suggest that the mean micelle/globule diameter (2*R*_s_) for the framboidal G_63_H_350_B_*z*_ vesicles increases from 34 nm to 76 nm as *z* is increased from 97 to 400. Similarly, SAXS analyses indicate that 2*R*_s_ increases from 36 nm to 85 nm for the same set of samples. However, it is worth emphasizing that only a few hundred globules were analyzed by TEM, whereas the SAXS data are averaged over many millions of globules, which ensures far more robust statistics. Some difference between micelle/globule diameters measured by TEM and SAXS is likely because SAXS interrogates partially hydrated globules in aqueous solution. In contrast, TEM is performed on dehydrated globules under ultrahigh vacuum conditions, which accounts for the marginally smaller dimensions in this case. Moreover, SAXS reports a volume-average diameter whereas TEM provides a number-average diameter, hence the former technique always oversizes relative to the latter.

The proposed structural model (Fig. 2a) does not account for the nanoscale phase separation between the PHPMA and PBzMA blocks which might be expected to occur during PBzMA growth (see Fig. 1). However, the difference between the scattering length densities of the copolymer components (*ξ*_PGMA_, *ξ*_PHPMA_ and *ξ*_PBzMA_) and water (*ξ*_H_2_O_) significantly exceeds the difference between the scattering length densities of the copolymer components alone (see ESI† for full details of the structural models used in the SAXS analysis). Thus SAXS is simply not sufficiently sensitive to confirm the phase separation between the PHPMA and PBzMA blocks that is responsible for the evolution in morphology from smooth vesicles to framboidal vesicles during the PISA synthesis. Thus, in order to scrutinize the anticipated phase separation between the PHPMA and PBzMA blocks, a contrast variation technique was employed in this study. Accordingly, the vesicle dispersions were prepared using a 40% w/w aqueous sucrose solution instead of water.

This solution is a good solvent for the PGMA stabilizer block and has a scattering length density of *ξ*_H_2_O+sucrose_ = 10.88 × 10^10^ cm^–2^, which lies between *ξ*_PHPMA_ and *ξ*_PBzMA_ (see ESI†). This contrast variation approach significantly reduces the scattering length density difference between the copolymer components and the continuous phase and consequently increases the sensitivity of SAXS towards the structural changes occurring within the vesicle membrane. It is emphasized that the PGMA stabilizer block has the highest scattering length density and hence produces a significant contribution to the scattering signal. Thus in principle contrast-matching the corona block (*ξ*_PGMA_ = 11.94 × 10^10^ cm^–2^) to the solvent would be informative, but unfortunately this was not possible because of the limited solubility of sucrose in water.

The contrast-matched copolymer dispersions were prepared in two steps: (1) preparation of a 44% w/w aqueous sucrose stock solution followed by (2) dilution of the copolymer dispersion prepared in pure water from 10% w/w to 1% w/w solids using this aqueous sucrose solution. The fitting parameters obtained for the purely aqueous dispersions were also used for SAXS analysis of the aqueous sucrose dispersions, while the solvent scattering length density used in the model was changed from that of water to that for 40% w/w aqueous sucrose solution. Assuming that the vesicle morphology and the copolymer concentration remain unchanged in the aqueous sucrose dispersion, only six parameters are required for the SAXS fitting: the membrane thickness corresponding to the parameters used for population 1, the spherical micelle radius corresponding to population 2, their corresponding standard deviations and relative concentrations of both populations. For each sample, the concentration ratio, *c*_2_/*c*_1_, was kept constant during the fitting at the same value obtained for the dispersions in pure water (Table S1†). This relatively constrained model produced satisfactory data fits for the SAXS patterns of the aqueous sucrose dispersions (see Fig. 2c, S4 and Table S1†). A significant inconsistency is only observed for the triblock copolymer prepared with the longest PBzMA block (Fig. S4,† see G_63_H_350_B_400_). In this case, including additional fitting parameters in the model associated with the spherical micelle packing (*R*_PY_ and *F*_PY_) and removing the *c*_2_/*c*_1_ ratio constraint produced a better data fit (Fig. S4,† solid red line). This latter fit indicated a significantly higher relative concentration for the second population (see the last entry in Table S1†). This suggests that these nano-objects are best described as strongly interacting (*i.e.* aggregated) spherical micelles, with little or no vesicular character. In general, SAXS analysis of this series of vesicles dispersed in aqueous sucrose solution demonstrates that both the vesicle membrane thickness and the mean micelle radius are slightly reduced relative to the corresponding values determined for the same vesicles dispersed in pure water. The lower degree of solvent plasticization results in a 15 % reduction in the membrane volume (see Table S1†). Presumably, this is simply because aqueous sucrose is a poorer solvent for the two blocks located in the membrane than water alone. Unfortunately, the relatively weak scattering from the aqueous sucrose dispersions means that SAXS pattern fits involving the other model parameters, including *x*_sol_, are considered unreliable. Nevertheless, the original SAXS model used for analysis of vesicle dispersions in pure water (Fig. 2a) was consistent with the SAXS patterns recorded for dispersions in aqueous sucrose solution.

In order to probe the nanoscale phase separation between the PHPMA and PBzMA blocks within the vesicle membrane, a more sophisticated two-population model composed of vesicles with a three-layer hydrophobic membrane and spherical core–shell–corona micelles was developed (see Fig. S5, eqn (S1) and (S11)–(S17)†). In this model, it is assumed that the PBzMA block occupies the central layer of the membrane. In principle, vesicles with the mean scattering length density of the hydrophobic component of the membrane that is closest to that of the aqueous sucrose solution (Table S1,† see G_63_H_350_B_125_ and G_63_H_350_B_169_) should be most sensitive to nanoscale phase separation. If there is a homogeneous distribution of PHPMA and PBzMA blocks within the membrane (continuous core model, see Fig. 2a), then the hydrophobic component of the membrane should barely contribute to the X-ray scattering as the difference between *ξ*_mc_ and *ξ*_H_2_O+sucrose_ is almost zero. Alternatively, if there is nanoscale phase separation between the PHPMA and PBzMA blocks (three-layer model, see Fig. S5†) the hydrophobic component of the membrane should produce a strong contribution to the scattering signal because of the significant difference between *ξ*_PHPMA_ and *ξ*_H_2_O+sucrose_ and between *ξ*_H_2_O+sucrose_ and *ξ*_PBzMA_. Given that phase separation between the PHPMA and PBzMA blocks should cause a redistribution of solvent concentration within the vesicle membrane, two scenarios for the sophisticated two-population model (eqn (S1), (S11) and (S14)†) were considered. As for the SAXS analyses summarized in Table S1,† in one scenario it is assumed that the solvent fraction in the PBzMA layer and two PHPMA layers of the membrane are equal (*i.e.*, *x*_PBzMAsol_ = *x*_PHPMAsol_ = 0.50). In an alternative scenario associated with Table S2,† it is assumed that *x*_PBzMAsol_ = 0 and *x*_PHPMAsol_ = 0.50. Comparison of SAXS patterns calculated for the continuous core (single-layer) model and these two more sophisticated three-layer models indicates that the continuous core model is actually more consistent with the experimental data (Fig. 2d).

To summarize the vesicle morphology studies, as the G_63_H_350_ diblock precursor is chain-extended with progressively longer PBzMA blocks, the overall vesicle diameter remains essentially constant (as indicated by DLS, TEM and SAXS) but the vesicle membrane thickness (as calculated by SAXS) increases. As a result, the vesicle lumen volume is gradually reduced on increasing the DP of the PBzMA. Finally, SAXS can be used to *quantify* the evolution in surface roughness indicated for these framboidal vesicles on the basis of TEM studies (see Fig. 1). For G_63_H_350_B_*z*_ triblock copolymer vesicles, both SAXS and TEM studies indicate that well-defined globules are only formed when *z* > 97 and the mean globule diameter increases monotonically from 36 nm (*z* = 97) to 85 nm (*z* = 400). However, a contrast variation approach used for SAXS analysis provides no evidence for the anticipated nanoscale phase separation between the hydrophobic PHPMA and PBzMA blocks within the membrane. This suggests that the PHPMA and PBzMA blocks may only be weakly segregated within the vesicle membrane, rather than strongly segregated (see Fig. 2d).

### Pickering emulsion studies

**Fig. 4:**
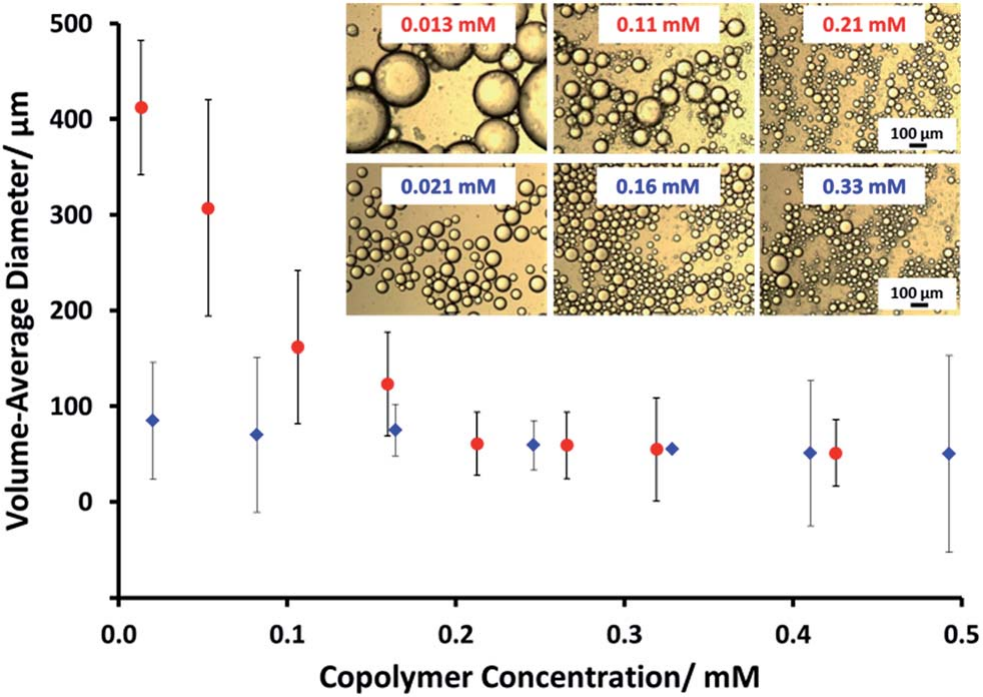
Volume-average diameter (determined for *n*-dodecane droplets by laser diffraction) *vs.* copolymer concentration for (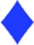) linear G_63_H_350_ diblock copolymer vesicles and (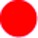) framboidal G_63_H_350_B_200_ triblock copolymer vesicles. Inset shows representative optical microscopy images for selected emulsions prepared at the stated copolymer concentrations. The scale bars are valid for all six images.

Framboidal G_63_H_350_B_200_ triblock copolymer vesicles (an intermediate PBzMA block length) and linear G_63_H_350_ diblock copolymer vesicles were each evaluated as putative Pickering emulsifiers for the stabilization of *n*-dodecane emulsion droplets in water. Aqueous vesicle dispersions (0.5% to 3.0% w/w) were homogenized with an equal volume of *n*-dodecane at 12 000 rpm for two minutes at 20 °C to produce Pickering emulsions. The concentration dependence of the mean droplet diameter of the resulting emulsions was determined by laser diffraction and optical microscopy (see Fig. 4). Increasing the concentration of linear G_63_H_350_ vesicles led to a constant mean droplet diameter of ∼70 μm. This suggests that the linear G_63_H_350_ vesicles do not withstand the high shear conditions required for emulsion preparation, and instead dissociate to produce individual copolymer chains, as previously reported by Thompson *et al.*^13^,^33^ In contrast, the mean emulsion droplet diameter prepared using the G_63_H_350_B_200_ triblock copolymer vesicles increases from 55 μm up to 412 μm over the same concentration range.

Similar concentration-dependent droplet diameters were observed for other G_63_H_350_B_*z*_ copolymer vesicles. These observations suggest that the G_63_H_350_B_*z*_ triblock copolymer vesicles survive high shear homogenization and consequently adsorb as intact triblock copolymer vesicles to produce genuine Pickering emulsions.

**Fig. 5:**
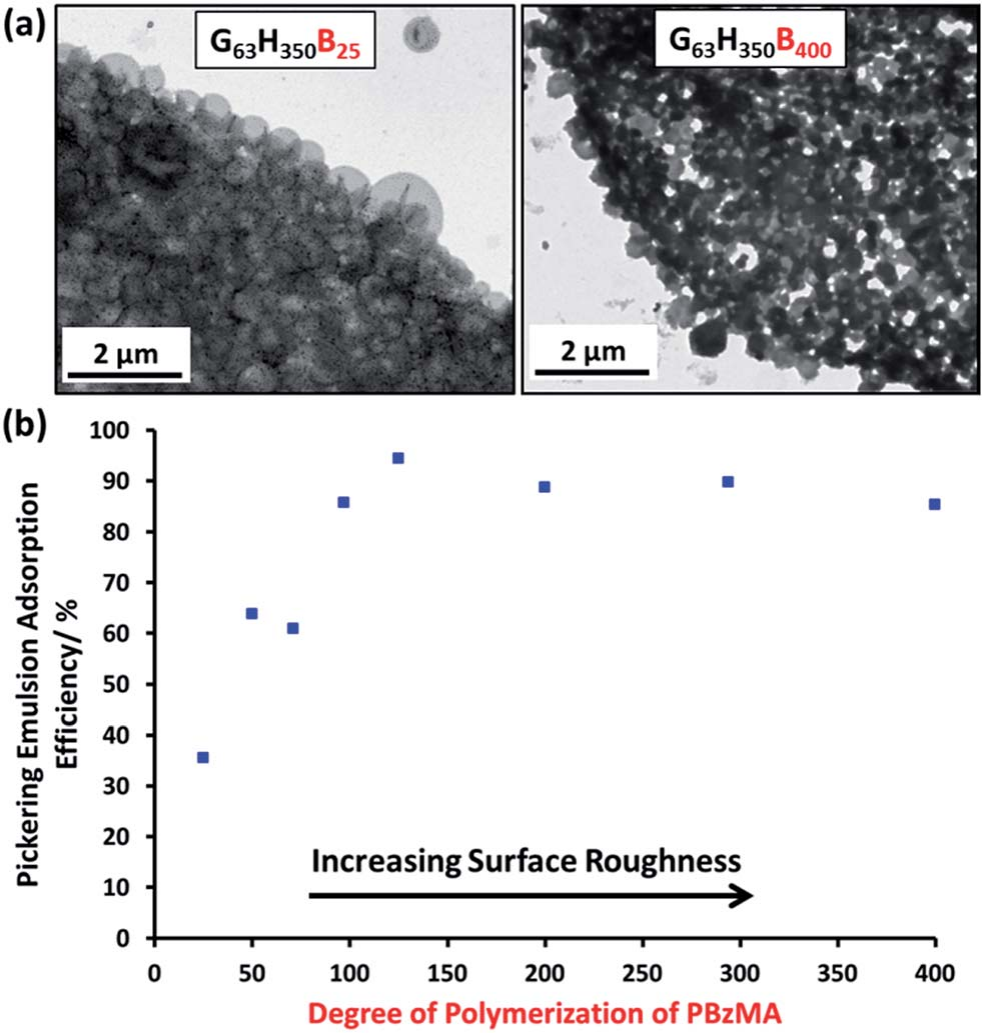
(a) TEM images obtained for Pickering emulsions of *n*-hexane stabilized by aqueous vesicle dispersions of G_63_H_350_B_25_ and G_63_H_350_B_400_ triblock copolymer vesicles. (b) Plot of *A*_eff_*vs.* PBzMA DP in a series of G_63_H_350_B_*z*_ triblock copolymer vesicles (0.20 mM) with increasing surface roughness.

Remarkably, only a relatively short PBzMA block is required to stabilize the vesicles during homogenization; presumably, the highly hydrophobic nature of this third block is sufficient to prevent vesicle dissociation. TEM (see Fig. 5a) and SEM studies (Fig. S6†) of the latter emulsions confirm that intact framboidal vesicles indeed act as Pickering emulsifiers. Hence the observed concentration dependence for the droplet diameter is readily explained: higher vesicle concentrations are required for stabilization of smaller oil droplets because of the concomitant increase in total surface area.

The Pickering emulsifier adsorption efficiency, *A*_eff_, was determined by turbidimetry experiments, as described by Thompson *et al.*^13^ First, scattering curves were recorded and calibration plots were constructed for each triblock copolymer vesicle evaluated (see Fig. S7†). The scattering intensity increased monotonically as the PBzMA DP is increased in the G_63_H_350_B_*z*_ triblock copolymer series, because of the significantly higher refractive index of this aromatic block. The Pickering emulsions proved to be highly stable towards coalescence, but creaming of the lower density droplet phase occurred on standing for 24 h at 20 °C. The turbidity of this lower aqueous phase was analyzed by visible absorption spectroscopy to determine the amount of vesicles remaining in the aqueous solution and hence the adsorbed amount by difference (see Table S3†). To confirm the validity of this turbidimetric assay, the vesicles were also sized by DLS before and after homogenization in order to ensure that no size fractionation occurred during vesicle adsorption at the oil/water interface.

At a copolymer concentration of 0.20 mM, the *A*_eff_ increased from 36% up to 94% on increasing the PBzMA DPs from 25 to 125 (see Fig. 5b). For PBzMA DPs greater than 125, the *A*_eff_ is progressively reduced, resulting in an *A*_eff_ of 85% at a mean DP of 400 (see Fig. 5b). These observations are similar to those reported by San-Miguel and Behrens,^15^ who observed that both the nanoparticle wettability and emulsion stability attained maximum values at the same root-mean-squared (rms) surface roughness. However, the latter parameter was calculated indirectly from AFM measurements performed on a planar surface that had been subjected to the same coating conditions as the spherical microparticles. Nevertheless, it was suggested that wetting of microparticles with up to 6 nm rms roughness occurred within the Wenzel regime,^34^ whereas the roughest microparticles (rms roughness = 7.5 nm) corresponded to the Cassie–Baxter regime.^35^ The former regime led to optimal Pickering emulsifier performance.

In the present study, the model *framboidal* vesicles exhibit substantially enhanced *A*_eff_ values compared to *non-framboidal* G_58_H_350_E_20_ cross-linked vesicles, for which a *A*_eff_ of 67% has been reported for a similar copolymer concentration.^13^ Presumably, the much higher surface roughness of the former nanoparticles (mean globule diameter ∼ 45 nm) is responsible for this observation. This is significantly different to the critical length scale reported by San-Miguel and Behrens.^15^ However, it seems likely that other parameters, *e.g.* charge *vs.* steric stabilization or differences in copolymer composition, also influence the particle contact angle (and hence surface wettability).

## Conclusions

G_63_H_350_ diblock copolymer precursor vesicles were chain-extended with BzMA *via* seeded RAFT emulsion polymerization at 70 °C to prepare a series of framboidal G_63_H_350_B_*z*_ triblock copolymer vesicles (where *z* ranged from 25 to 400). TEM images reveal that the vesicle surface becomes increasingly pitted and rough until individual PBzMA globules can be observed protruding from the membrane. As higher PBzMA DPs are targeted, these globules gradually increase in size and become more prominent. SAXS provides a more in-depth analysis of surface roughness compared to TEM. Both SAXS and TEM studies confirm that topologically smooth vesicles are obtained prior to chain extension with BzMA, after which the vesicles acquire framboidal character (and hence surface roughness) depending on the DP of the PBzMA. A two-population SAXS model has been developed in order to characterize the globules protruding from the vesicle membrane. The mean globule diameter increases monotonically from 36 nm to 85 nm when the diblock copolymer precursor is chain-extended with 97–400 units of BzMA. Unlike the G_63_H_350_ diblock copolymer precursor vesicles, the framboidal triblock copolymer vesicles survive high shear homogenization conditions and can therefore act as Pickering emulsifiers for the stabilization of *n*-dodecane droplets. Turbidimetry data support the literature hypothesis that greater surface roughness does indeed promote higher Pickering emulsifier adsorption efficiencies. More specifically, framboidal vesicles with mean globule dimensions of 45 nm exhibit a *A*_eff_ of up to 94%. PISA represents a highly convenient and versatile synthetic route to colloidal particles of exquisitely tunable surface roughness. Such nanoparticles may also be of interest for other fundamental scientific studies, such as the effect of surface topology on cell uptake kinetics.^36^

## Experimental

### Materials and methods

#### Materials

All reagents were used as received unless otherwise stated. Benzyl methacrylate (BzMA), *n*-dodecane and 4,4′-azobis-4-cyanopentanoic acid (ACVA) were purchased from Sigma-Aldrich (UK). BzMA inhibitor was removed by passing this monomer through an inhibitor removal column. Ethanol, dichloromethane, DMSO and DMF were purchased from Fisher Scientific (UK). Glycerol monomethacrylate (GMA) was kindly donated by GEO Specialty Chemicals (Hythe) and used without further purification. 2-Hydroxypropyl methacrylate (HPMA) was purchased from Alfa Aesar (UK) and contained 0.07 mol% dimethacrylate impurity, as judged by high performance liquid chromatography (HPLC). CD_3_OD and d_6_-DMSO NMR solvents were purchased from Goss Scientific (UK). 4-Cyano-4-(2-phenylethanesulfanylthiocarbonyl)sulfanyl-pentanoic acid (PETTC) was synthesized in-house.^37^ Deionized water was obtained using an Elga Elgastat Option 3A water purifier; its pH was approximately 6.2 and its surface tension was 72.0 mN m^–1^ at 20 °C.

#### RAFT synthesis of PGMA macro-CTA agent in ethanol

A round-bottomed flask was charged with GMA (30.0 g; 187 mmol), PETTC (1.01 g; 2.97 mmol), ACVA (167 mg, 0.156 mmol) and ethanol (39.5 g). The sealed reaction vessel was purged with N_2_ for 30 min and placed in a pre-heated oil bath at 70 °C for 135 min. The resulting PGMA macro-CTA (GMA conversion = 87%; *M*_n_ = 17 600 g mol^–1^, *M*_w_/*M*_n_ = 1.16) was purified by precipitation into excess dichloromethane. A mean DP of 63 was calculated for this macro-CTA using ^1^H NMR spectroscopy by comparison of the integral from 3.4 ppm to 4.3 ppm due to five protons from the PGMA with that of the peaks around 7 ppm due to the five aromatic protons from the RAFT CTA (see Fig. S2†).

#### Preparation of linear PGMA–PHPMA diblock copolymer precursor vesicles *via* RAFT aqueous dispersion polymerization at 15% w/w solids

PGMA_63_ macro-CTA (5.00 g, 0.485 mmol), HPMA monomer (24.5 g, 170 mmol) and deionized water (167 g, purged with N_2_ for 30 min) were weighed into a 250 mL round-bottomed flask and purged with N_2_ for 20 min. ACVA was added (68.9 mg, 0.242 mmol, CTA/ACVA molar ratio = 2.0) and purged with N_2_ for a further 10 min prior to immersion in an oil bath set at 70 °C for 2 h. Finally, the polymerization was quenched by cooling to room temperature with subsequent exposure to air.

#### Preparation of PGMA–PHPMA–PBzMA triblock copolymer vesicles *via* RAFT seeded emulsion polymerization at 10–19% w/w solids

PGMA_63_–PHPMA_350_ diblock precursor vesicles (15.0 mL of a 10% w/w copolymer dispersion, 1.50 g copolymer, 0.0247 mmol), ACVA (1.38 mg, 0.00494 mmol, CTA/ACVA molar ratio = 5.0) and BzMA monomer (0. 109 g, 0.617 mmol, target DP = 25) were weighed into a 40 mL sample vial and purged with N_2_ for 20 min prior to immersion in an oil bath set at 70 °C for 4 h. Then the polymerization was quenched by cooling to room temperature and subsequent exposure to air. A series of similar copolymer syntheses were performed for which the PBzMA target DP ranged from 50 to 400 using BzMA masses varying from 0.218 g to 1.74 g (1.23 mmol to 9.87 mmol), respectively.

#### Pickering emulsion formation

*n*-Dodecane (2.0 mL) was homogenized with 2.0 mL of a 0.5–3.0% w/v aqueous vesicle dispersion for 2 min using a IKA Ultra-Turrax T-18 homogenizer with a 10 mm dispersing tool operating at 12 000 rpm. The droplets were imaged by OM and the mean droplet diameter was assessed by laser diffraction.

#### Turbidimetry experiments

Pickering emulsions were allowed to cream overnight before an appropriate amount of the aqueous phase was extracted and diluted ten-fold, before measuring the absorbance from 400 to 800 nm using visible absorption spectroscopy. Calibration plots were constructed for each vesicle dispersion by recording the absorbance at 750 nm of the vesicle dispersions, varying the copolymer concentration from 0.00625 to 0.1% w/w.

### Characterization

#### ^1^H NMR spectroscopy

All NMR spectra were recorded using a 400 MHz Bruker Avance-400 spectrometer and 64 scans were averaged per spectrum. The mean DP of the PBzMA block was calculated as described previously by Chambon *et al.*^24^

#### Gel permeation chromatography (GPC)

Copolymer molecular weights and polydispersities were determined using a DMF GPC set-up operating at 60 °C and comprising two Polymer Laboratories PL gel 5 μm Mixed C columns connected in series to a Varian 390 LC multi-detector suite (only the refractive index detector was utilized) and a Varian 290 LC pump injection module. The GPC eluent was HPLC grade DMF containing 10 mM LiBr at a flow rate of 1.0 mL min^–1^. DMSO was used as a flow-rate marker. Calibration was conducted using a series of ten near-monodisperse poly(methyl methacrylate) standards (*M*_n_ = 625–618 000 g mol^–1^). The chromatograms were analyzed using Varian Cirrus GPC software (version 3.3) provided by the instrument manufacturer (Polymer Laboratories).

#### Dynamic light scattering (DLS)

Intensity-average hydrodynamic diameters of the copolymer dispersions were determined using a Malvern Zetasizer NanoZS instrument. Dilute aqueous dispersions (0.10% w/w) were analyzed using disposable cuvettes and all data were averaged over three consecutive runs to give the hydrodynamic diameter (*D*_h_).

#### Transmission electron microscopy (TEM)

Aggregate solutions were diluted fifty-fold at 20 °C to generate 0.10% w/w dispersions. Copper/palladium TEM grids (Agar Scientific) were surface-coated in-house to yield a thin film of amorphous carbon. The grids were then plasma glow-discharged for 30 s to create a hydrophilic surface. Individual samples (0.1% w/w, 12 μL) were adsorbed onto the freshly glow-discharged grids for 1 min and then blotted with filter paper to remove excess solution. To stain the aggregates, uranyl formate (0.75% w/v) solution (9 μL) was soaked on the sample-loaded grid for 20 s and then carefully blotted to remove excess stain. The grids were then dried using a vacuum hose. Imaging was performed on a Phillips CM100 instrument at 100 kV, equipped with a Gatan 1k CCD camera.

#### Small-angle X-ray scattering (SAXS)

SAXS patterns were recorded at two synchrotron sources (ESRF, station ID02, Grenoble, France and Diamond Light Source, station I22, Didcot, UK). A monochromatic X-ray radiation (wavelength *λ* = 0.995 Å and 1.001 Å, respectively) and 2D SAXS detectors (FReLoN Kodak CCD and Pilatus 2M, respectively) were used for these experiments. The SAXS camera length set-ups covered the *q* range from 0.0009 Å^–1^ to 0.004 Å^–1^ (ESRF) and from 0.002 Å^–1^ to 0.19 Å^–1^ (Diamond), where 
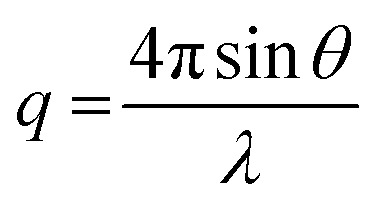
 is the modulus of the scattering vector and *θ* is one-half of the scattering angle. Either a 2.0 mm diameter glass capillary (ESRF) or a liquid cell composed of two mica windows (each of 25 μm thickness) separated by a polytetrafluoroethylene spacer of 1 mm thickness (Diamond) were used as sample holders, respectively. X-ray scattering data were reduced by Nika SAS data reduction macros for Igor Pro (integration, normalization, background subtraction) and were further analyzed using Irena SAS macros for Igor Pro. SAXS measurements were conducted on G_63_H_350_B_*z*_ (*z* = 0–400, see Table S1†) dispersions either in water (ESRF and Diamond) or in a 40% w/w aqueous sucrose solution (Diamond). The copolymer concentration was diluted from 10% w/w (as-synthesized) to 1.0% w/w for data collection.

#### Visible absorption spectroscopy

Turbidities of both the initial vesicle dispersions and also the underlying aqueous phase of the corresponding creamed emulsions after homogenization with *n*-dodecane were assessed by visible absorption spectrophotometry (Perkin-Elmer Lambda 25 instrument) between 400 and 800 nm at a scan speed of 960 nm min^–1^.

#### Optical microscopy (OM)

Optical microscopy images were recorded using a Motic DMBA300 digital biological microscope with a built-in camera and equipped with Motic Images Plus 2.0 ML software.

#### Laser diffraction

A Malvern Mastersizer 2000 instrument equipped with a small volume Hydro 2000SM sample dispersion unit (*ca.* 50 mL), a He–Ne laser operating at 633 nm, and a solid-state blue laser operating at 466 nm was used to size each emulsion. The stirring rate was adjusted to 1000 rpm in order to avoid creaming of the emulsion during analysis. After each measurement, the cell was rinsed once with ethanol, followed by three rinses with doubly-distilled water; the glass walls of the cell were carefully wiped with lens cleaning tissue to avoid cross-contamination and the laser was aligned centrally to the detector prior to data acquisition. The volume-average diameter was measured and repeated four times for each emulsion.

## Supplementary Material

Supplementary information
