# Supplementary material for: Framboidal ABC triblock copolymer vesicles: a new class of efficient Pickering emulsifier
Source: Chem Sci. 2015 Aug 5;6(11):6179–88. doi: 10.1039/c5sc02346g (PMC6054058; doi:10.1039/c5sc02346g)
Supplement: Supplementary file 1 [file SC-006-C5SC02346G-s001.pdf]

Electronic Supplementary Material (ESI) for Chemical Science.
This journal is © The Royal Society of Chemistry 2015

Supporting Information for Chemical Sciencemanuscript:

Framboidal ABC Triblock Copolymer Vesicles: A New Class of Efficient Pickering Emulsifier

Charlotte J. Mable, Nicholas J. Warren, Kate L. Thompson, Oleksandr O. Mykhaylyk, Steven P. Armes*

STRUCTURAL MODELS FOR SAXS ANALYSIS

Three distinctive copolymer morphologies could be identified by TEM studies (see Figure 1): (morphology 1, M1) vesicles with smooth featureless membranes, (morphology 2, M2) vesicles with pitted surfacesand (morphology 3, M3) vesicles with particulate membranescomposed of an assembly of small micelle-like spheres (Figure 2a). The latter morphology is comparable to core-shell nanocomposite particles (comprising polymer cores and particulate silica shells) reported by Balmer and co-workers[1-4], who demonstrated that the corresponding SAXS patterns could be fitted using a two-population model obtained by combining a core-shell model with a particulate shell.[1] A similar approach has been undertaken in this work. In the following description of the SAXS models, the hydrophobic component of the vesicle membrane (i.e., the PHPMA and PBzMA blocks) is called the membrane (or a membrane core) and the hydrophilic component of the vesicle membrane (i.e., the PGMA block) is called the corona.

In general, the scattering intensity of a system composed of *n* different (non-interacting) populations of polydisperse objects can be expressedas:

$$I(q) = \sum_{l=1}^{n} S_l(q) N_l \int_0^\infty \dots \int_0^\infty F_l(q, r_{l1}, \dots, r_{lk})^2 \Psi_l(r_{l1}, \dots, r_{lk}) dr_{l1} \dots dr_{lk} \quad \text{S1}$$

Where $F_l(q, r_{l1}, \dots, r_{lk})$ is the form factor, $\Psi_l(r_{l1}, \dots, r_{lk})$ is the distribution function, N_l is the number density per unit volume and $S_l(q)$ is the structure factor of the lth population in the system. $r_{l1},\dots,r_{lk}$ is a set of *k* parameters describing the structural morphology of the lth population. Based on our previous studies,[3, 4] it was assumed that the SAXS patterns can be represented as a sum of scattering signals generated by two populations (*n* = 2 in Equation S1): (i) vesicles (the first population, *l* = 1 in Equation S1) and (ii) spherical micelles forming the vesicle membrane (the second population, *l* = 2 in Equation S1). In the case of vesicles with smooth (or pitted) membranes, the second population can be simply removed from the model (*n* = 1 in Equation S1). The form factor for the first population, represented by vesicles, can be described as:[5]

$$F_1(q) = [A_{mc1}(q)]^2 + N_{agg1}\beta_c^2 F_c(qR_g) + N_{agg1}(N_{agg1}-1)\beta_c^2[A_{c1}(q)]^2 + 2N_{agg1}\beta_c A_{mc1}(q)A_{c1}(q) \quad \text{S2}$$

The amplitude of the membrane self-term in Equation S2 is:

$$A_{mc1}(q) = (1 - x_{sol})(\xi_{mc} - \xi_{sol})[V_{out}\Phi(qR_{mcout}) - V_{in}\Phi(qR_{mcin})]\exp\left(-\frac{q^2\sigma_{in}^2}{2}\right) \quad \text{S3}$$

where $R_{mcin} = R_{mc} - \frac{1}{2}T_{mc}$ is the inner radius of the membrane, $R_{mcout} = R_{mc} + \frac{1}{2}T_{mc}$ is the outer radius of the membrane, $V_{in} = \frac{4}{3}\pi R_{mcin}^3$ and $V_{out} = \frac{4}{3}\pi R_{mcout}^3$. R_{mc} is the radius from the centre of the vesicle to the middle of the membrane and T_{mc} is the membrane thickness (see Figure 2a). $\Phi(x) = \frac{3[\sin(x) - x\cos(x)]}{(x)^3}$ is the form factor amplitude of a sphere. The exponent term represents a sigmoidal interface (width σ_{in}) that describes a decaying scattering length density between the PBzMA and PHPMA membrane-forming blocks and the PGMA corona. This parameter was fixed in the model at 0.25 nm. The vesicle aggregation number (i.e. the mean number of copolymer chains per vesicle) is given by $N_{agg1} = (1 - x_{sol})(V_{out} - V_{in}) / V_{mc}$, where x_{sol} is the solvent fraction in the membrane and V_{mc} is the volume of the membrane block components ($V_{mc} = V_{PHPMA350} + V_{PBzMAz}$). In this model it is assumed that the hydrophobic PHPMA and PBzMA blocks are uniformly distributed within the membrane. The corona block X-ray scattering length contrast is $\beta_c = V_c(\xi_c - \xi_{sol})$, where V_c is the corona block volume (V_{PGMA63}). The block volumes are calculated from $V = \frac{M_n}{N_A \rho}$ (Table S1) using the number-average molecular weight, M_n, of the block components and the mass densities of the three blocks comprising the copolymer ($\rho_{PHPMA} = 1.21 \pm 0.01$ g cm^{-3}, $\rho_{PBzMA} = 1.15 \pm 0.01$ g cm^{-3} and $\rho_{PGMA} = 1.31 \pm 0.01$ g cm^{-3}; these values were determined for the corresponding homopolymers using a helium pycnometer). ξ_{mc}, ξ_c, and ξ_{sol} are the X-ray scattering length densities of the membrane [$\xi_{mc} = (V_{PHPMA350}\xi_{PHPMA} + V_{PBzMAz}\xi_{PBzMA})/V_{mc}$, where $\xi_{PHPMA} = 11.11 \times 10^{10}$ cm^{-2} and $\xi_{PBzMA} = 10.41 \times 10^{10}$ cm^{-2}], the vesicle corona block ($\xi_{PGMA} = 11.94 \times 10^{10}$ cm^{-2}) and the solvent ($\xi_{H2O} = 9.42 \times 10^{10}$ cm^{-2} for water or $\xi_{H2O+sucrose} = 10.88 \times 10^{10}$ cm^{-2} for the 40 % w/w aqueous sucrose solution assuming that scattering length density of sucrose is 14.36×10^{10} cm^{-2} and mass density of water and sucrose is 1.0 g cm^{-3} and 1.59 g cm^{-3}, respectively). The self-correlation term of the corona block is given by the Debye function $F_c(qR_g) = \frac{2[\exp(-q^2R_g^2) - 1 + q^2R_g^2]}{q^4R_g^4}$, where R_g is the radius of gyration of the corona block. Assuming that there is no penetration of the corona blocks within the membrane, the amplitude of the corona self-term is expressed as:

$$A_{c1}(q) = \psi(qR_g)\frac{1}{2}\left[\frac{\sin[q(R_{mcout} + R_g)]}{q(R_{mcout} + R_g)} + \frac{\sin[q(R_{mcin} - R_g)]}{q(R_{mcin} - R_g)}\right] \quad \text{S4}$$

where $\psi(qR_g) = \dfrac{1-\exp(-qR_g)}{(qR_g)^2}$ is the factor amplitude of the corona chain. The polydispersities for two parameters (R_{mc} and T_{mc}), expressed as a Gaussian distribution, are considered for the first (vesicle) population:

$$\Psi_1(r_{11}, r_{12}) = \frac{1}{\sqrt{2\pi\sigma_{Rmc}^2}} e^{-\frac{(r_{11}-R_{mc})^2}{2\sigma_{Rmc}^2}} \frac{1}{\sqrt{2\pi\sigma_{Tmc}^2}} e^{-\frac{(r_{12}-T_{mc})^2}{2\sigma_{Tmc}^2}} \quad \text{S5}$$

whereσ_{Rmc} and σ_{Tmc} are the standard deviations forR_{mc} and T_{mc}, respectively. The number density per unit volume of the first population (l = 1 in Equation S1) is expressed as:

$$N_1 = \frac{c_1}{\int_0^\infty \int_0^\infty V_1(r_{11}, r_{12}) \Psi_1(r_{11}, r_{12}) dr_{11} dr_{12}} \quad \text{S6}$$

wherec_1 is the total *volume fraction* of copolymer in the vesicles and $V_1(r_{11}, r_{12})$ is the total *volume* of copolymers in a vesicle [$V_1(r_{11}, r_{12}) = (V_{mc} + V_c) N_{agg1}(r_{11}, r_{12})$]. A dilutevesicle dispersion is assumed in the model, so the structure factor of the first population is set to unity [$S_1(q) = 1$]. The first population describes scattering from a continuous membrane (Figure 2a, smooth vesicles). If the membrane has a particulate sub-structure, this generatesan additional scattering signal that can be described by a second population (Figure 2a, spherical micelles), l = 2 in Equation S1). The form factor of this population can be represented by a spherical micelle model:[5]

$$F_2(q) = [A_{mc2}(q)]^2 + N_{agg2}\beta_c^2 F_c(qR_g) + N_{agg2}(N_{agg2}-1)\beta_c^2 [A_{c2}(q)]^2 + 2N_{agg2}\beta_c A_{mc2}(q) A_{c2}(q) \quad \text{S7}$$

If not stated otherwise, the parameters and functions in this micelle model are analogous to those of the vesicle model, EquationS2. The amplitude of the core self-term in Equation S7is $A_{mc2}(q) = (1-x_{sol})(\xi_{mc} - \xi_{sol}) \frac{4}{3}\pi R_s^3 \Phi(qR_s) \exp\left(-\frac{q^2\sigma_{in}^2}{2}\right)$, where R_s is the radius of the spherical micelle core (Figure 2a). The form factor amplitude of the spherical micelle corona is $A_{c2}(q) = \psi(qR_g) \dfrac{\sin[q(R_s + R_g)]}{q(R_s + R_g)}$ where no penetration of the corona chains within the micelle cores was assumed. As for the vesicle model, β_c corresponds to the X-ray scattering length contrast of the corona block. The aggregation number, N_{agg2}, for the spherical micelles is given by $N_{agg2} = (1-x_{sol}) \dfrac{4\pi R_s^3}{3V_{mc}}$. The polydispersity for one parameter (R_s) can be expressed as a Gaussian distribution and is considered for the second population of spherical micelles:

$$\Psi_2(r_{21}) = \frac{1}{\sqrt{2\pi\sigma_{Rs}^2}} e^{-\frac{(r_{21}-R_s)^2}{2\sigma_{Rs}^2}} \qquad \text{S8}$$

whereσ_{Rs} is the standard deviation forR_s. The number density per unit volume of the second population (l = 2 in Equation S1) is expressed as:

$$N_2 = \frac{c_2}{\int_0^\infty V_2(r_{21})\Psi_2(r_{21})dr_{21}} \qquad \text{S9}$$

wherec_2 is the total *volume fraction* of copolymer in the spherical micelles and $V_2(r_{21})$ is the total *volume* of copolymer in a spherical micelle [$V_2(r_{21}) = (V_{mc} + V_c)N_{agg2}(r_{21})$]. An effective structure factor expression previously proposed for interacting spherical micelles[6]is used for the second population in order to describe dense packing of the spherical micelles within the vesicle membrane:

$$S_2(q) = 1 + \frac{A_{mic}(q)^2[S_{PY}(q, R_{PY}, f_{PY}) - 1]}{F_2(q)} \qquad \text{S10}$$

Herein the form factor of the average radial scattering length density distribution of micelles is $A_{mic}(q) = A_{mc2}(q) + N_{agg2}\beta_c A_{c2}(q)$ and $S_{PY}(q, R_{PY}, f_{PY})$ is a hard-sphere interaction structure factor based on the Percus-Yevick approximation[7], where R_{PY} is the interaction radius ($R_{PY} = R_s + \Delta R_{PY}$, ΔR_{PY} is a fitting parameter) and F_{PY} is the hard-sphere volume fraction. A numerical integration was used for EquationS1, EquationS6 and EquationS9 during the fitting.

Nanoscale phase separation between the hydrophobic PBzMA and PHPMA blocks was also considered and avesicle model with a three-layer membrane structure was developed for the corresponding SAXS analysis (see Figure 2d and Figure S4). In this model it is assumed that phase-separated blocks of PBzMA and PHPMA occupy both the central layer of the membrane and also anadjacent layer. Again, a two-population approach (n = 2 in Equation S1) was used and, by analogy to Equation S2,the vesicle form factor corresponding to the first population can be expressed as:

$$F_1(q) = [A_{mc1}^{3l}(q)]^2 + N_{agg1}^{3l}\beta_c^2 F_c(qR_g) + N_{agg1}^{3l}(N_{agg1}^{3l} - 1)\beta_c^2[A_{c1}(q)]^2 + 2N_{agg1}^{3l}\beta_c A_{mc1}^{3l}(q)A_{c1}(q) \qquad \text{S11}$$

In this case the amplitude of the membrane self-term is

$$\begin{aligned} A_{mc1}^{3l}(q) = \{&(1 - x_{PHPMAsol})(\xi_{PHPMA} - \xi_{sol})[V_{out}\Phi(qR_{mcout}) - V_{PBzMAout}\Phi(qR_{PBzMAout})] + \\ &+ (1 - x_{PBzMAsol})(\xi_{PBzMA} - \xi_{sol})[V_{PBzMAout}\Phi(qR_{PBzMAout}) - V_{PBzMAin}\Phi(qR_{PBzMAin})] + \\ &+ (1 - x_{PHPMAsol})(\xi_{PHPMA} - \xi_{sol})[V_{PBzMAin}\Phi(qR_{PBzMAin}) - V_{in}\Phi(qR_{mcin})]\} \exp\left(-\frac{q^2\sigma_{in}^2}{2}\right) \end{aligned} \qquad \text{S12}$$

where $R_{PBzMAin} = R_{mc} - \frac{1}{2}T_{PBzMA}$ is the inner radius of the PBzMA layer of the membrane, $R_{PBzMAout} = R_{mc} + \frac{1}{2}T_{PBzMA}$ is the outer radius of the PBzMA layer, $V_{PBzMAin} = \frac{4}{3}\pi R^3_{PBzMAin}$ and $V_{PBzMAout} = \frac{4}{3}\pi R^3_{PBzMAout}$. Because the number of PBzMA blocks and PHPMA blocks occupying the three-layer membrane must be the same and equal to the aggregation number, N^{3l}_{agg1}, then it follows that T_{PBzMA} is defined from the following relation:

$$\frac{4}{3}\pi[(R_{mc} + \frac{1}{2}T_{PBzMA})^3 - (R_{mc} - \frac{1}{2}T_{PBzMA})^3]\frac{(1 - x_{PBzMAsol})}{V_{PBzMAy}} =$$
$$\frac{4}{3}\pi[(R_{mc} + \frac{1}{2}T_{mc})^3 - (R_{mc} + \frac{1}{2}T_{PBzMA})^3 + (R_{mc} - \frac{1}{2}T_{PBzMA})^3 - (R_{mc} - \frac{1}{2}T_{mc})^3]\frac{(1 - x_{PHPMAsol})}{V_{PHPMA}}$$

where$x_{PBzMAsol}$ and $x_{PHPMAsol}$ are the solvent fraction in the PBzMA layer and both PHPMA layers of the membrane, respectively. The vesicle aggregation number is given by: $N^{3l}_{agg1} = (1 - x_{PHPMAsol})[V_{out} - V_{in} - (V_{PBzMAout} - V_{PBzMAin})]/V_{PHPMA350}$. The other parameters utilized for thisthree-layermembrane vesicle model (Equation S11) have the same definitions as those used in the single-layer membrane vesicle model (Equation S2). The number density per unit volume of the first population (l = 1 in Equation S1) is expressed as:

$$N_1 = \frac{c_1}{\int_0^\infty\int_0^\infty V_1^{3l}(r_{11}, r_{12})\Psi_1(r_{11}, r_{12})dr_{11}dr_{12}} \qquad \text{S13}$$

wherec_1 is the total *volume fraction* of copolymer in population 1and $V_1^{3l}(r_{11}, r_{12})$ is thecorresponding total *volume* of copolymer [$V_1^{3l}(r_{11}, r_{12}) = (V_{PBzMAz} + V_{PHPMA350} + V_c)N^{3l}_{agg1}(r_{11}, r_{12})$]. Polydispersities for two parameters (R_{mc} and T_{mc}) areintroduced for the first population (Equation S5). A dilute vesicle dispersion is assumed, so the structure factor of the first population can be set to unity [$S_1(q) = 1$].The form factor for thesecond population of particles associated with the particulate membrane structure, l = 2 in Equation S1, can be represented by a spherical micelle model where the core is represented by two components as a result of the nanoscale phase separation of the PHPMA and PBzMA blocks (Figure S5). By analogy to EquationS7, we have:

$$F_2(q) = [A^{3l}_{mc2}(q)]^2 + N_{agg2}\beta_c^2 F_c(qR_g) + N_{agg2}(N_{agg2} - 1)\beta_c^2[A_{c2}(q)]^2 + 2N_{agg2}\beta_c A^{3l}_{mc2}(q)A_{c2}(q) \text{ S}$$

The amplitude of the core self-term is given by:

$$A_{mc2}^{3l}(q) = [(1 - x_{PHPMAsol})(\xi_{PHPMA} - \xi_{sol})V_s\Phi(qR_s) + \\ + (1 - x_{PBzMAsol})(\xi_{PBzMA} - \xi_{PHPMA})V_{sin}\Phi(qR_{sin})]\exp\left(-\frac{q^2\sigma_{in}^2}{2}\right) \quad \text{S15}$$

where R_{sin} is the inner radius of the PHPMA shell (i.e. the PBzMA core radius of the micelles) and R_s is the outer radius of the PHPMA shell, such that $V_{sin} = \frac{4}{3}\pi R_{sin}^3$ and $V_s = \frac{4}{3}\pi R_s^3$. If not stated otherwise, parameters and functions in this model are the same as those used in the vesicle model, Equation S12.The aggregation number for the spherical micelles is given by $N_{agg2}^{3l} = (1 - x_{PHPMAsol})(V_s - V_{sin}) / V_{PHPMA350}$. Because the number of PBzMA blocks and PHPMA blocks occupying the micelle core must be the same and equal to the aggregation number, N_{agg2}^{3l}, R_{sin} is defined from the following relation:

$$\frac{4}{3}\pi R_{sin}^3 \frac{(1 - x_{PBzMAsol})}{V_{PBzMAz}} = \frac{4}{3}\pi(R_s^3 - R_{sin}^3)\frac{(1 - x_{PHPMAsol})}{V_{PHPMA}}$$

The other parameters used in this spherical micelle model with phase-separated micelle cores (Equation S14) have the same definition as those used in the spherical micelle model (Equation S7). The number density per unit volume of the second population is expressed as:

$$N_2 = \frac{c_2}{\int_0^\infty V_2^{3l}(r_{21})\Psi_2(r_{21})dr_{21}} \quad \text{S16}$$

where c_2 is the total *volume fraction* of copolymer in population 2and $V_2^{3l}(r_{21})$ is thecorresponding total *volume* of copolymer [$V_2^{3l}(r_{21}) = (V_{PBzMAz} + V_{PHPMA350} + V_c)N_{agg2}^{3l}(r_{21})$]. The polydispersity for one parameter (R_s)is considered for the second population (EquationS8). An effective structure factor expression proposed for interacting spherical micelles[6]was used for the second population in order to describe the dense micelle packing within the vesicle membrane:

$$S_2(q) = 1 + \frac{A_{mic}^{3l}(q)^2[S_{PY}(q, R_{PY}, f_{PY}) - 1]}{F_2(q)} \quad \text{S17}$$

Herein the form factor of the average radial scattering length density distribution of the micelles is given by $A_{mic}^{3l}(q) = A_{mc2}^{3l}(q) + N_{agg2}^{3l}\beta_c A_{c2}(q)$. Numerical integration was used for EquationS13 and EquationS16 during the fitting.

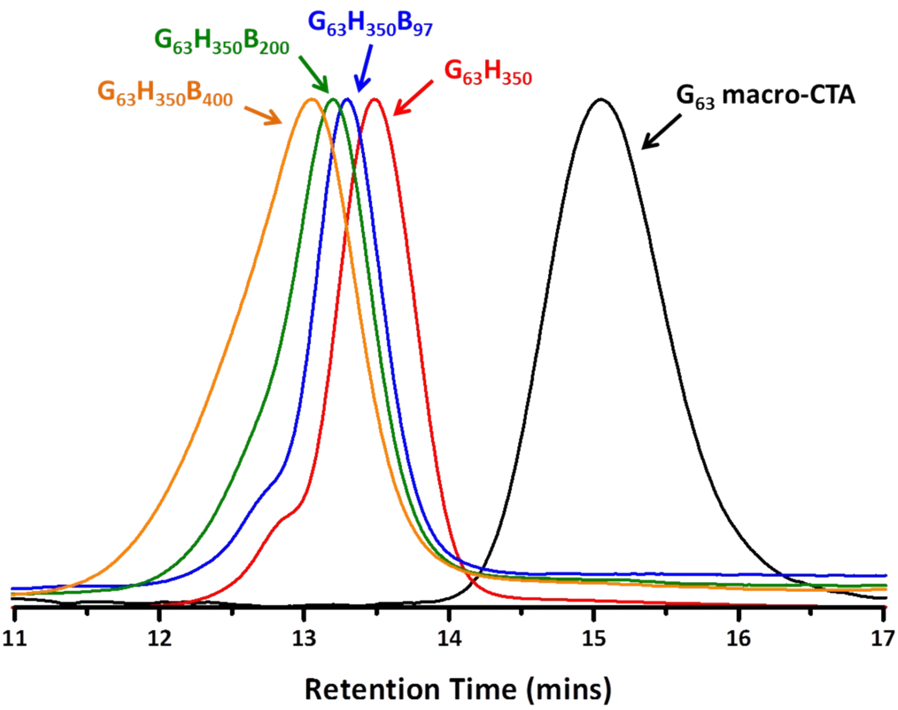

Figure S1. DMF GPC curves (vs. PMMA standards) obtained for the G_{63} macro-CTA, linear $G_{63}H_{350}$ diblock copolymer precursor, and selected $G_{63}H_{350}B_z$ triblock copolymers (where z ranges from 25 to 400). Number-average molecular weights and polydispersities obtained for all nine of these copolymers are shown in Table 1**.**

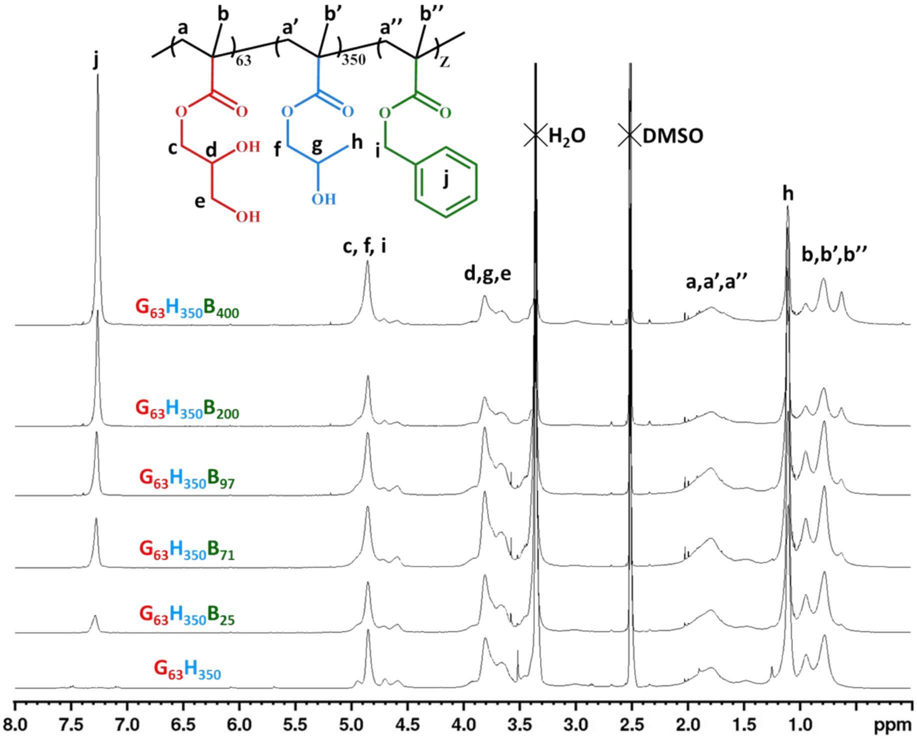

Figure S2. Assigned ^{1}H NMR spectra (d_6-DMSO) obtained for the $G_{63}H_{350}$ linear diblock copolymer precursor and five of the corresponding $G_{63}H_{350}B_z$triblock copolymers (where z ranges from 25 to 400).

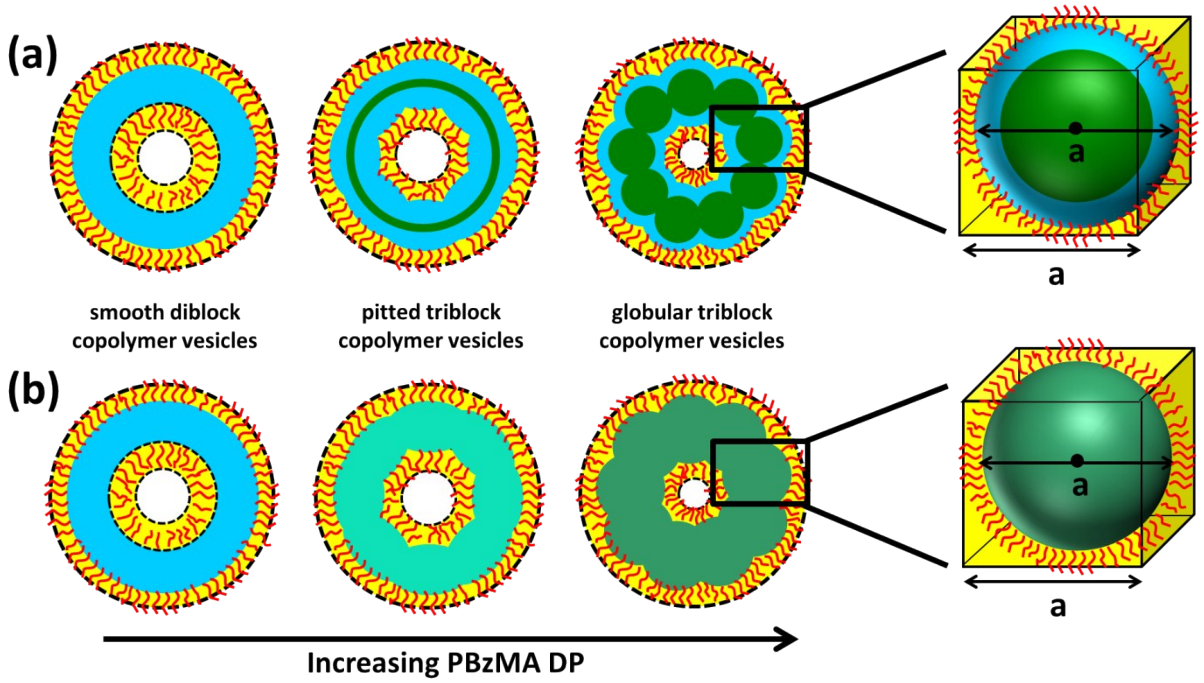

Figure S3. Schematicillustration of the effect of varying the PBzMA DP (z) on the value of x_{sol} for $G_{63}H_{350}B_z$ vesicles. (a) The originally predicted phase-separated three-layer cross-section(the SAXS model is described by Equations S1, S11 and S14), where red = PGMA, blue = PHPMA and green = PBzMA. (b) Schematic representation of the smooth, pitted and framboidal vesicles described by the SAXS model assuming that the hydrophobic PHPMA and PBzMA blocks are uniformly distributed within the membrane(Equations S1, S2 and S7),where red = PGMA, light blue = PHPMA, light green = mixed PHPMA plus PBzMA membrane where $z \leq 50$, and dark green = mixed PHPMA plus PBzMA membrane where $z \geq 97$. In each case, the yellow areas indicate the presence of water between adjacent globules. The inset cubes indicate the approximate volume occupied by each globule within the vesicle membrane.

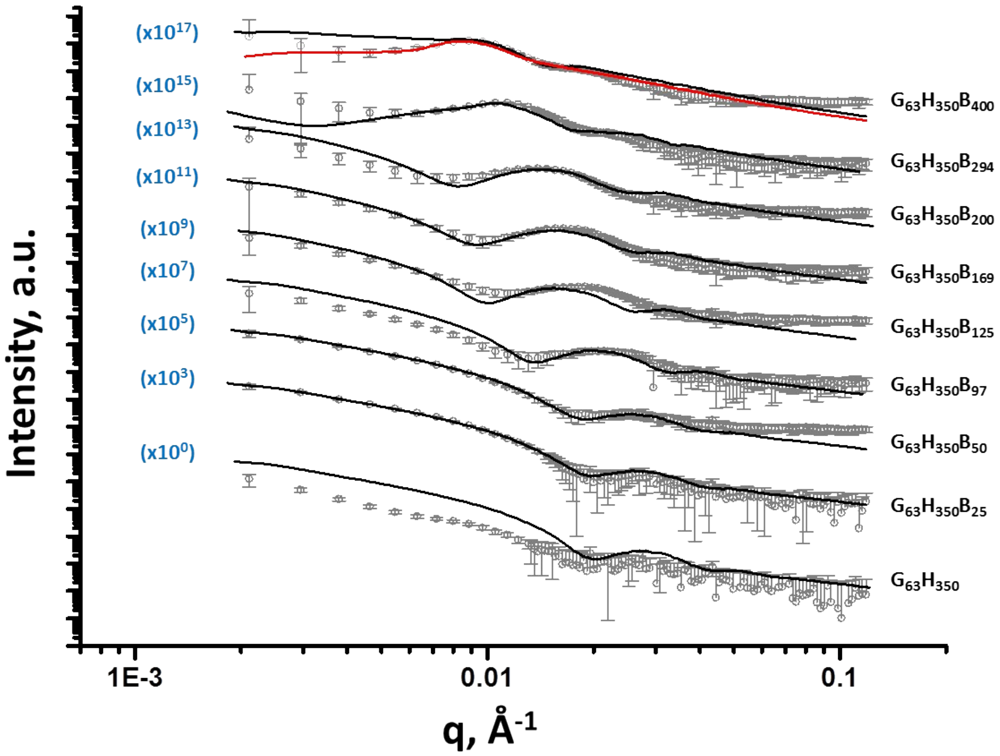

Figure S4. SAXS patterns and data fits obtained for a 1.0 % w/w dispersion of $G_{63}H_{350}$ diblock copolymer precursor vesicles in 40 % w/w aqueous sucrose and eight framboidal $G_{63}H_{350}B_z$ triblock copolymer vesicles, where z ranges from 25 to 400. Solid black lines represent data fitsassuming that the hydrophobic PHPMA and PBzMA blocks are uniformly distributed within the membrane (Figure 2a): the first three vesicle samples were analyzed assuming a singlevesicle population(Equation S1, n = 1), but for vesicles with a PBzMA DP of 97 or above a two-population model was required for satisfactory data fits(Equation S1, n = 2). The solid red line represents the data fit obtained for $G_{63}H_{350}B_{400}$ triblock copolymer vesicles when a different set of fitting parameters were used based on an aggregated sphere model for which c_2/c_1 was allowed to vary (see Table S1). The SAXS patterns are shifted upward by an arbitrary factor, which is displayed in blue.

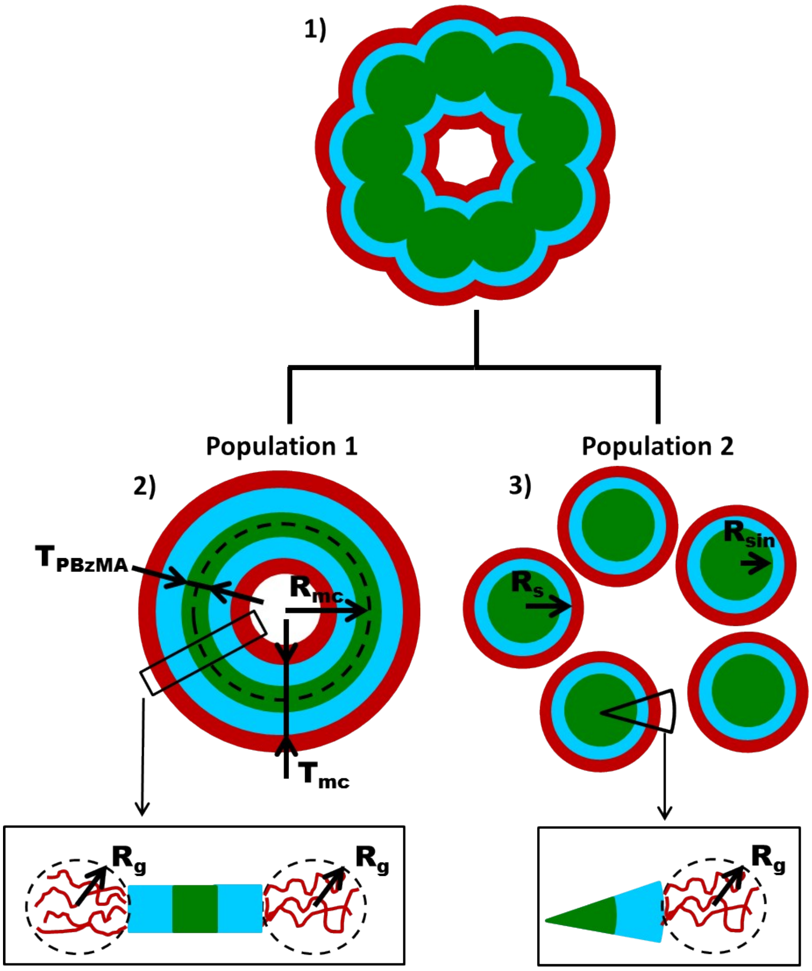

Figure S5. Schematic representation illustrating the three-layer, phase-separated membrane model (red = PGMA, blue = PHPMA and green = PBzMA) used to fit SAXS data, where (1) is a cartoon showing a three-layer vesicle model for which the following two-population model is required;(2) represents population 1, which is a vesicle model comprising a three-layer membrane, and (3) represents population 2, which is a spherical micelle model (core-shell-corona).

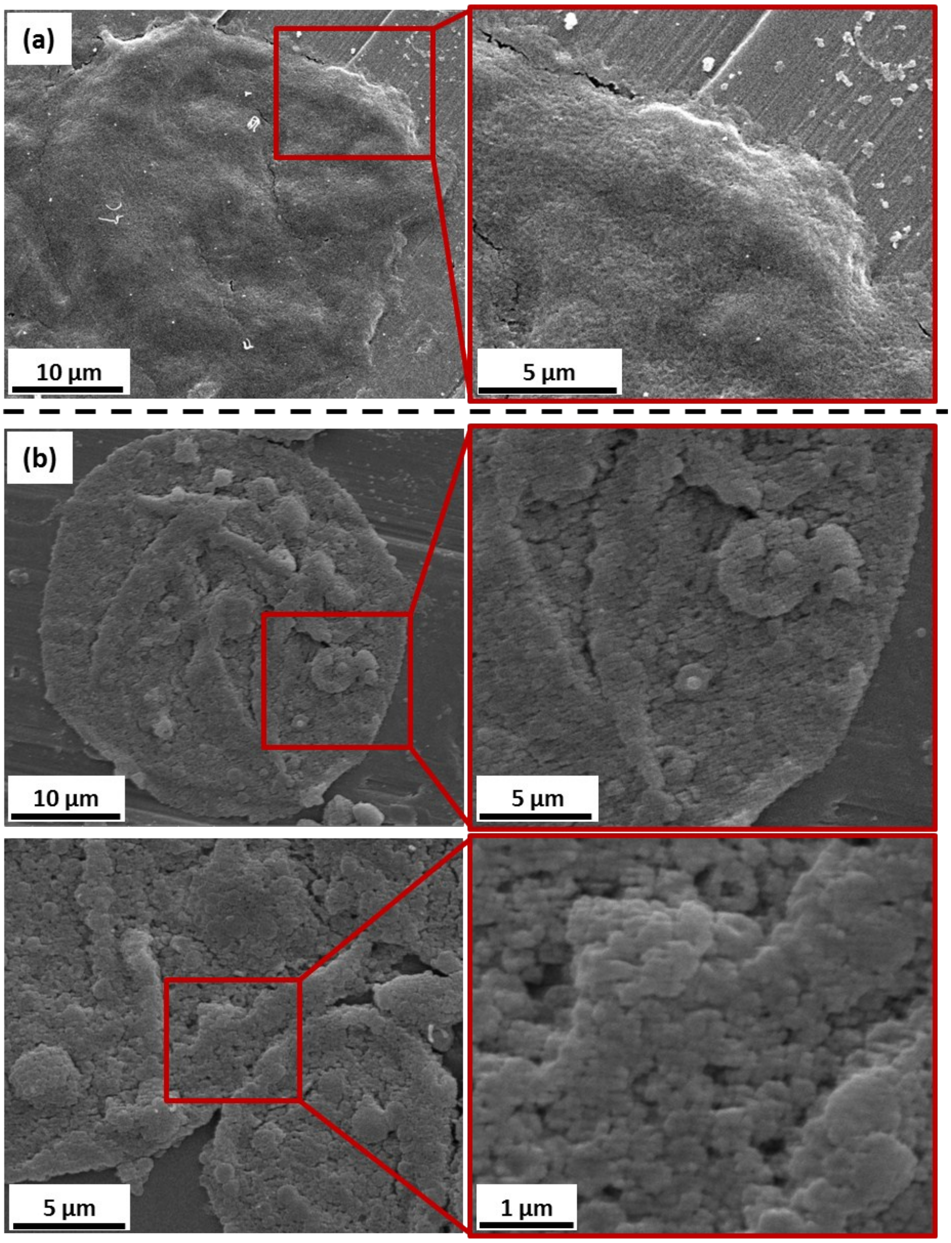

Figure S6. SEM images obtained for oil-in-water Pickering emulsions stabilized by dispersions of (a) *non-framboidal*$G_{63}H_{350}$ diblock copolymer vesicles and (b) *framboidal* $G_{63}H_{350}B_{400}$ triblock copolymer vesicles. The images show that the former $G_{63}H_{350}$ vesicles dissociate during high shear homogenization, leading to the formation of an emulsion stabilized by individual diblock copolymer chains, rather than a genuine Pickering emulsion. In contrast, the latter *framboidal*vesicles proved to be stable when subjected to the same emulsification conditions and hence a genuine Pickering emulsion is produced.

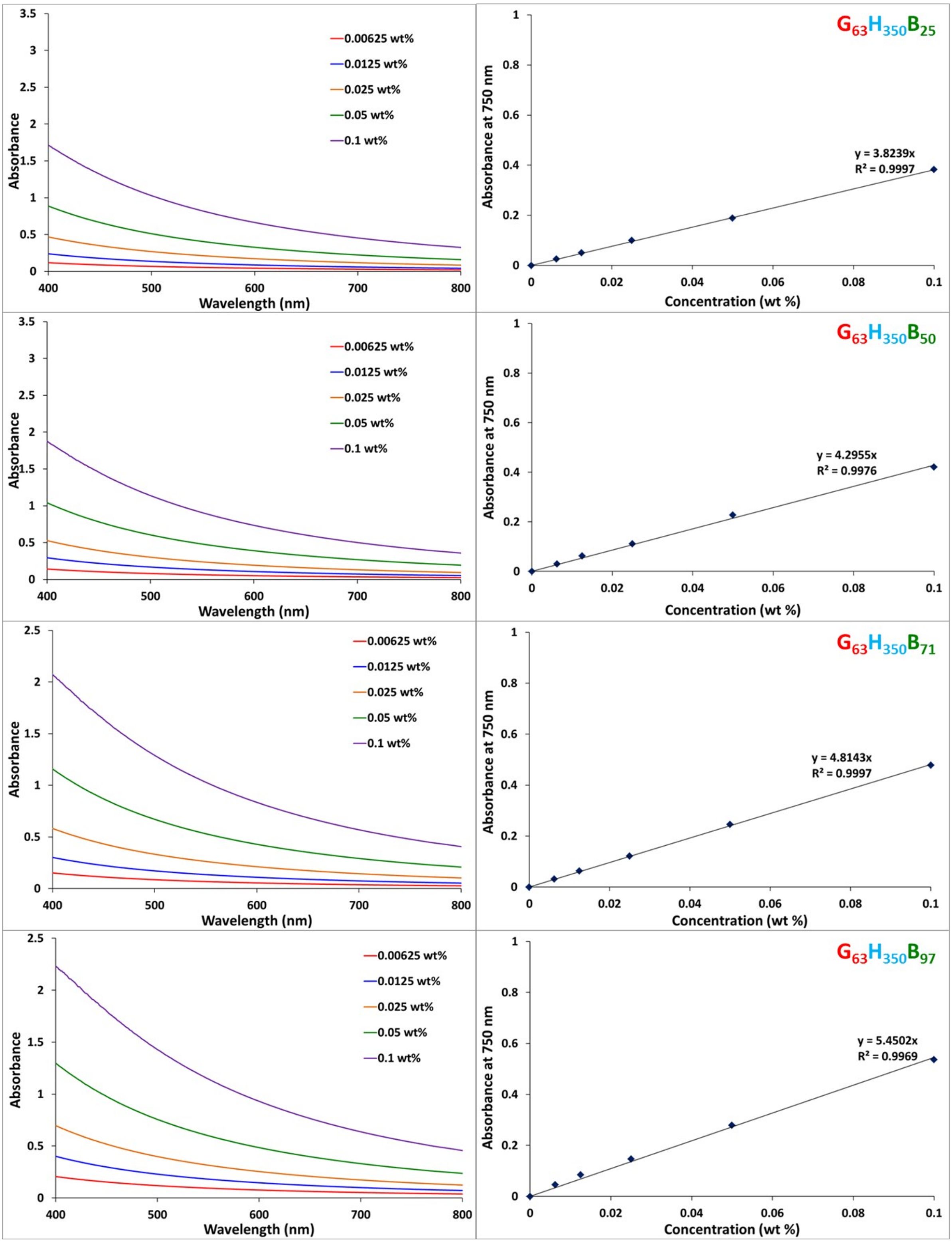

Absorbance
Wavelength (nm)
0.00625 wt%
0.0125 wt%
0.025 wt%
0.05 wt%
0.1 wt%
Absorbance at 750 nm
Concentration (wt %)
G63H350B25
y = 3.8239x
R² = 0.9997
G63H350B50
y = 4.2955x
R² = 0.9976
G63H350B71
y = 4.8143x
R² = 0.9997
G63H350B97
y = 5.4502x
R² = 0.9969

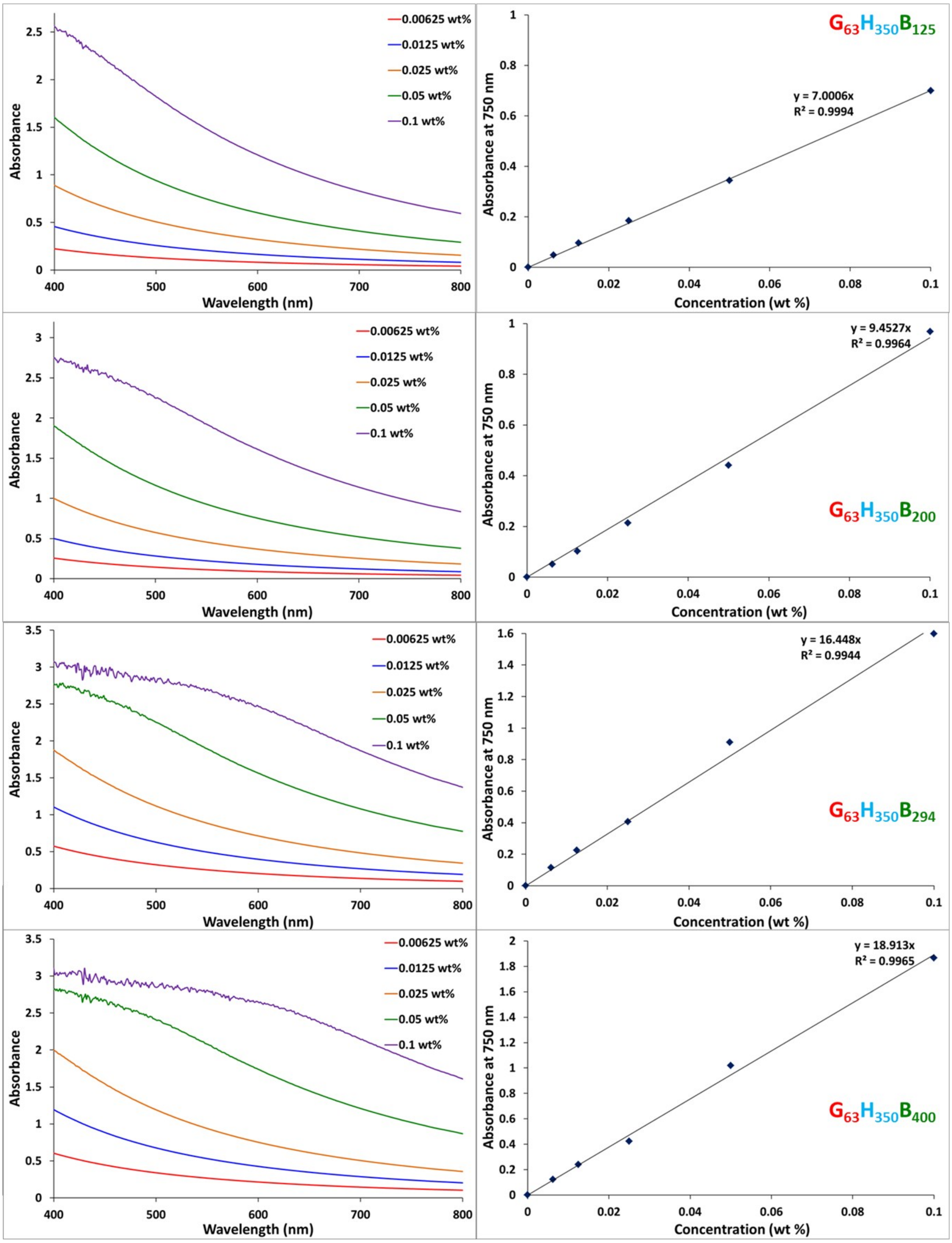

Figure S7. Visible absorption spectra (left) recorded for a series of $G_{63}H_{350}B_z$copolymer vesicles (where z ranges from 25 to 400) at various copolymer concentrations. An arbitrary wavelength (750 nm) was used to construct a linear calibration plot (right), which allows the concentration of non-adsorbed vesicles remaining in the aqueous phase after emulsification to be determined via turbidimetry.

Table S1. Structural parameters obtained for a series of $G_{63}H_{350}B_z$ (z = 0 to 400)copolymer dispersions in either pure water or a 40 % w/w aqueous sucrose solution from SAXS analysis using the two-population model (EquationS1, n = 2). Representative parameters of the first population model (population 1) corresponding to vesicles (whose form factor is described by Equation S2): R_{mc} is the radius from the center of the vesicle to the center of the membrane (Figure 2a) and $\sigma_{R\mathrm{mc}}$ is the associated standard deviation, T_{mc} is the membrane core thickness and $\sigma_{T\mathrm{mc}}$ is the associated standard deviation, R_{out} is the total radius of the vesicle ($R_{out} = R_{mc} + \frac{1}{2}T_{mc} + 2R_g$, where the radius of gyration of the corona PGMA block, R_g, is 2.1 nm - see the main text). Representative parameters of the second population (population 2) corresponding to spherical micelles (whose form factor is described by Equation S7): R_s is the core radius, σ_{Rs} is the standard deviation of the core radius, R_{PY} is the Percus-Yevick correlation radius of densely-packed spherical micelles and F_{PY} is the Percus-Yevick effective volume fraction of the packed micelles. c_2/c_1 is the ratio of the volume fraction of the copolymers forming spherical micelles (population 2) to the volume fraction of the copolymers forming vesicles (population 1). V_{PBzMA} denotes the volume of the hydrophobic PBzMA block. Two sets of fitting parameters are given for $G_{63}H_{350}B_{400}$ in a 40 % w/w aqueous sucrose solution.

Code	Sample	V_{PBzMA}/nm^3	ξ_{mc} ×10^{-10} /cm^{-2}	Population 1					c_2/c_1	Population 2			
				R_{mc} (σ_{Rmc}) /nm	T_{mc} (σ_{Tmc})/ nm		R_{out}/ nm			R_s (σ_{Rs})/ nm		R_{PY} /nm	F_{PY} /nm
					Water	Sucrose	Water	Sucrose		Water	Sucrose		
CM42	$G_{63}H_{350}$	-	11.11	163(47)	18.9(2.5)	18.9(2.5)*	176	176	-	-	-	-	-
CM43	$G_{63}H_{350}B_{25}$	6.378	11.05	155(46)	21.8(3.1)	18.2(2.7)	170	168	-	-	-	-	-
CM44	$G_{63}H_{350}B_{50}$	12.76	11.00	156(45)	23.1(3.5)	18.8(4.1)	172	169	-	-	-	-	-
CM46	$G_{63}H_{350}B_{97}$	24.75	10.93	155(44)	26.7(3.7)	24.8(3.3)	172	171	0.132	18.7(3.4)	18.7(3.4)*	15.8	0.35
CM47	$G_{63}H_{350}B_{125}$	31.89	10.89	155(43)	30.6(4.4)	26.6(4.3)	174	173	0.223	22.3(4.0)	22.3(4.0)*	20.7	0.43
CM49	$G_{63}H_{350}B_{169}$	43.11	10.84	156(42)	34.2(5.2)	27.2(3.9)	177	174	0.343	24.7(3.8)	22.7(4.3)	21.8	0.40
CM50	$G_{63}H_{350}B_{200}$	51.02	10.81	154(43)	37.8(6.3)	30.7(5.0)	177	173	0.425	26.9(3.6)	25.5(2.1)	22.6	0.36
CM51	$G_{63}H_{350}B_{294}$	75.00	10.75	145(46)	45.5(7.6)	35.7(4.6)	172	167	0.575	34.9(4.1)	30.9(3.8)	31.9	0.47
CM52	$G_{63}H_{350}B_{400}$	102.0	10.69	142(44)	54.8(8.1)	44.0(10.7)	173	168	0.612	43.2(6.0)	36.7(5.2)	40.2	0.50
		102.0	10.69	142(44)	-	41.3(10.8)	-	167	20.3	-	34.5(6.3)	30.8	0.43

* These data were not considered to be reliable

Table S2. Structural parameters obtained for a series of $G_{63}H_{350}B_z$ (z = 0 to 400)copolymer dispersions in either pure water or a 40 % w/w aqueous sucrose solution from SAXS analysis using the two-population model (EquationS1, *n* = 2). Representative parameters of the first population model (population 1) corresponding to vesicles (whose form factor is described by Equation S2): x_{sol} is the volume fraction of solvent in the hydrophobic part of the vesiclemembrane, R_{mc} is the radius from the center of the vesicle to the center of the membrane (Figure 2a) and σ_{Rmc} is the associated standard deviation, T_{mc} is the membrane core thickness and σ_{Tmc} is the associated standard deviation, $R_{out}$is the total radius of the vesicle ($R_{out} = R_{mc} + \frac{1}{2}T_{mc} + 2R_g$, where the radius of gyration of the corona PGMA block, R_g, is 2.1 nm - see the main text). Representative parameters of the second population (population 2) corresponding to spherical micelles (whose form factor is described by EquationS7): R_s is the core radius, σ_{Rs} is the standard deviation of the core radius, R_{PY} is the Percus-Yevick correlation radius of densely-packed spherical micelles and F_{PY} is the Percus-Yevick effective volume fraction of the packed micelles. c_2/c_1 is the ratio of the volume fraction of the copolymers forming micelles (population 2) to the volume fraction of the copolymers forming vesicles (population 1). V_{PBzMA} denotes the volume of the hydrophobic PBzMA block.

**Sample**	**V_{PBzMA}/nm³**	**ξ_{mc} ×10⁻¹⁰ /cm⁻²**	**X_{sol}**	**Population 1**					**c_2/c_1**	**Population 2**			
				**R_{mc} (σ_{Rmc}) /nm**	**T_{mc} (σ_{Tmc})/ nm**		**R_{out}/ nm**			**R_s (σ_{Rs})/ nm**		**R_{PY} /nm**	**F_{PY} /nm**
					**Water**	**Sucrose**	**Water**	**Sucrose**		**Water**	**Sucrose**		
$G_{63}H_{350}$	-	11.11	0.5	163(47)	18.9(2.5)	18.9(2.5)*	176	176	-	-	-	-	-
$G_{63}H_{350}B_{25}$	6.378	11.05	0.46	155(46)	21.9(3.1)	18.3(2.8)	170	168	-	-	-	-	-
$G_{63}H_{350}B_{50}$	12.76	11.00	0.42	156(45)	23.2(3.5)	18.7(4.1)	172	169	-	-	-	-	-
$G_{63}H_{350}B_{97}$	24.75	10.93	0.37	155(44)	26.3(3.6)	24.8(3.1)	172	171	0.158	14.3(5.1)	14.3(5.1)*	10.4	0.31
$G_{63}H_{350}B_{125}$	31.89	10.89	0.34	155(43)	30.4(4.9)	26.3(3.5)	174	173	0.321	23.3(3.6)	20.8(4.0)*	20.9	0.49
$G_{63}H_{350}B_{169}$	43.11	10.84	0.31	156(42)	33.9(5.6)	27.0(4.4)	177	174	0.388	25.2(3.6)	22.7(4.3)	21.8	0.42
$G_{63}H_{350}B_{200}$	51.02	10.81	0.29	154(43)	37.2(7.0)	30.7(5.3)	177	173	0.452	27.3(3.3)	25.7(2.5)	22.4	0.39
$G_{63}H_{350}B_{294}$	75.00	10.75	0.24	145(46)	45.0(8.0)	36.1(4.4)	172	167	0.611	34.5(3.7)	31.5(3.9)	30.1	0.46
$G_{63}H_{350}B_{400}$	102.0	10.69	0.20	142(44)	54.3(8.9)	43.4(15.7)	173	169	0.693	42.5(6.0)	36.6(2.8)	38.1	0.46
					-	46.0 (10.7)	-	169	1.818	-	36.2 (6.1)	31.8	0.46

* These data were not considered to be reliable.

Table S3. Determination of the adsorbed concentration of $G_{63}H_{350}B_z$ triblock copolymer vesicles (z = 0 to 400)as determined by analysis of the lower aqueous phase of the creamed emulsions.

	Initial Copolymer Concentration (wt. %)	Initial Copolymer Concentration (mM)	Copolymer Concentration in the Aqueous Phase (wt. %)*	Copolymer Concentration Adsorbed (wt. %)	Adsorption Efficiency (%)
**$G_{58}H_{350}B_{25}$**	0.5	0.08	0.28	0.22	44
	1.0	0.15	0.65	0.35	35
	1.5	0.23	0.97	0.53	36
	2.0	0.31	1.66	0.34	17
	2.5	0.39	2.26	0.24	10
	3.0	0.46	2.59	0.41	14
**$G_{58}H_{350}B_{50}$**	0.5	0.07	0.06	0.44	88
	1.0	0.15	0.35	0.65	65
	1.5	0.22	0.54	0.96	64
	2.0	0.29	1.23	0.77	38
	2.5	0.37	1.91	0.59	24
	3.0	0.44	2.51	0.49	16
**$G_{58}H_{350}B_{71}$**	0.5	0.07	0.16	0.34	67
	1.0	0.14	0.45	0.55	55
	1.5	0.21	0.58	0.92	61
	2.0	0.28	1.12	0.88	44
	2.5	0.35	1.43	1.07	43
	3.0	0.43	2.51	0.49	16
**$G_{58}H_{350}B_{97}$**	0.5	0.07	0.03	0.47	94
	1.0	0.13	0.31	0.69	69
	1.5	0.20	0.21	1.29	86
	2.0	0.26	0.63	1.37	69
	2.5	0.33	1.05	1.45	58
	3.0	0.40	2.11	0.89	30
**$G_{58}H_{350}B_{125}$**	0.5	0.06	0.00	0.50	100
	1.0	0.12	0.03	0.97	97
	1.5	0.18	0.08	1.42	94
	2.0	0.24	0.19	1.81	90
	2.5	0.30	0.23	2.27	91
	3.0	0.36	0.87	2.13	71
**$G_{58}H_{350}B_{200}$**	0.5	0.05	0.03	0.47	93
	1.0	0.11	0.06	0.94	94
	1.5	0.16	0.11	1.39	93
	2.0	0.21	0.22	1.78	89
	2.5	0.27	0.41	2.09	84
	3.0	0.32	0.94	2.06	69
**$G_{58}H_{350}B_{294}$**	0.5	0.05	0.01	0.49	99
	1.0	0.09	0.03	0.97	97
	1.5	0.14	0.07	1.43	95
	2.0	0.18	0.20	1.80	90
	2.5	0.23	0.41	2.09	83
	3.0	0.27	0.95	2.05	68
**$G_{58}H_{350}B_{400}$**	0.5	0.04	0.00	0.50	100
	1.0	0.08	0.02	0.98	98
	1.5	0.12	0.09	1.41	94
	2.0	0.16	0.21	1.79	90
	2.5	0.20	0.37	2.13	85
	3.0	0.24	0.81	2.19	73

*As determined by turbidimetry from a linear Beer-Lambert plot of absorbance (at an arbitrary fixed wavelength of 750 nm) against vesicle concentration.

1. J. A. Balmer, O. O. Mykhaylyk, J. P. A. Fairclough, A. J. Ryan, S. P. Armes, M. W. Murray, K. A. Murray and N. S. J. Williams, *Journal of the American Chemical Society*, 2010, **132**, 2166-2168.
2. J. A. Balmer, O. O. Mykhaylyk, A. Schmid, S. P. Armes, J. P. A. Fairclough and A. J. Ryan, *Langmuir*, 2011, **27**, 8075-8089.
3. L. A. Fielding, O. O. Mykhaylyk, S. P. Armes, P. W. Fowler, V. Mittal and S. Fitzpatrick, *Langmuir*, 2012, **28**, 2536-2544.
4. L. A. Fielding, O. O. Mykhaylyk, A. Schmid, D. Pontoni, S. P. Armes and P. W. Fowler, *Chemistry of Materials*, 2014, **26**, 1270-1277.
5. J. Bang, S. Jain, Z. Li, T. P. Lodge, J. S. Pedersen, E. Kesselman and Y. Talmon, *Macromolecules*, 2006, **39**, 1199-1208.
6. J. S. Pedersen, *Journal of Chemical Physics*, 2001, **114**, 2839-2846.
7. D. J. Kinning and E. L. Thomas, *Macromolecules*, 1984, **17**, 1712-1718.
